# Challenges and opportunities for falls prevention: an online survey across European healthcare professionals

**DOI:** 10.1007/s41999-025-01237-5

**Published:** 2025-06-17

**Authors:** Lotta J. Seppala, James Frith, Dawn A. Skelton, Clemens Becker, Hubert Blain, Rose-Anne Kenny, Annemiek J. Linn, Jesper Ryg, Solveig A. Arnadottir, Gülistan Bahat, Maria Bonnici, María Ángeles Caballero Mora, Yannis Dionyssiotis, Dvora Frankenthal, Sirpa Hartikainen, Jorunn L. Helbostad, Alvaro Casas Herrero, Birkan İlhan, Anna B. Jonsdottir, Marija Markovski, Regina Roller-Wirnsberger, Carmelinda Ruggiero, Ingvild Saltvedt, Anna Skalska, Daniel Smedberg, George Soulis, Katarzyna Szczerbińska, Eva Topinkova, Gregor Veninšek, Ellen Vlaeyen, Alban Ylli, Nathalie van der Velde, the European Geriatric Medicine Society Special Interest Group on Falls and Fractures

**Affiliations:** 1 Department of Internal Medicine, Section of Geriatric Medicine, Amsterdam UMC Location University of Amsterdam, Amsterdam, The Netherlands; 2 Amsterdam Public Health Research Institute, Amsterdam, The Netherlands; 3 The Newcastle Upon Tyne Hospitals NHS Foundation Trust, Newcastle Upon Tyne, NE1 4LP UK; 4 Population Health Science Institute, Newcastle University, Newcastle Upon Tyne, NE2 4AX UK; 5 School of Health and Life Sciences, Research Centre for Health (ReaCH), Glasgow Caledonian University, Cowcaddens Road, Glasgow, Scotland, UK; 6 Department of Clinical Gerontology and Geriatric Rehabilitation, Robert Bosch Hospital, Stuttgart, Germany; 7 Department of Geriatrics, Montpellier University Hospital and MUSE, Montpellier, France; 8 Department of Medical Gerontology, School of Medicine, Trinity College Dublin and Mercers Institute for Successful Ageing, St James’s Hospital, Dublin, Ireland; 9 Department of Communication, Amsterdam School of Communication Research/ASCoR, University of Amsterdam, Amsterdam, The Netherlands; 10 Department of Geriatric Medicine, Odense University Hospital, Odense, Denmark; 11 Geriatric Research Unit, Department of Clinical Research, University of Southern Denmark, Odense, Denmark; 12 Department of Physiotherapy, Faculty of Medicine, School of Health Sciences, University of Iceland, Reykjavik, Iceland; 13 Division of Geriatrics, Department of Internal Medicine, Istanbul Medical Faculty, Istanbul University, Capa, Istanbul, Turkey; 14 Department of Geriatric Medicine, Karin Grech Rehabilitation Hospital, Pietà, Malta; 15 Mater Dei Hospital l-Imsida, Malta & St Vincent De Paul, Luqa, Malta; 16 Hospital General Universitario de Ciudad Real, Ciudad Real, Spain; 17 2nd Physical Medicine and Rehabilitation Department, National Rehabilitation Center EKA, Athens, Greece; 18 Israel Center for Disease Control (ICDC), Ministry of Health, Ramat-Gan, Israel; 19 School of Pharmacy, Faculty of Health Sciences, University of Eastern Finland, Kuopio, Finland; 20 Department of Neuromedicine and Movement Science, Faculty of Medicine and Health Sciences, Norwegian University of Science and Technology, Trondheim, Norway; 21 Geriatric Department, Hospital Universitario de Navarra (HUN), Pamplona, Spain; 22 Universidad Pública de Navarra (UPNA), IdiSNA, Pamplona, Spain; 23 CIBER of Frailty and Healthy Aging (CIBERFES), Instituto de Salud CarlosIII, Madrid, Spain; 24 Division of Geriatrics, Department of Internal Medicine, Liv Hospital Vadistanbul, Istanbul, Turkey; 25 Department of Geriatric Medicine, Landspitali University Hospital, Reykjavík, Iceland; 26 Faculty of Medicine, University of Iceland, Reykjavík, Iceland; 27 Department of Physical Medicine and Rehabilitation, General Hospital 8Mi Septemvri, Skopje, North Macedonia; 28 Department of Internal Medicine, Medical University of Graz, Auenbruggerplatz 15, 8036 Graz, Austria; 29 Division of Gerontology and Geriatrics, Department of Medicine and Surgery, University of Perugia, Geriatric and Orthogeriatric Units, Azienda Ospedaliera di Perugia, Perugia, Italy; 30 Department of Neuromedicine and Movement Science, Faculty of Medicine and Health Science, NTNU – Norwegian University of Science and Technology, Trondheim, Norway; 31 Department of Geriatric Medicine, Clinic of Medicine, St. Olavs Hospital, Trondheim University Hospital, Trondheim, Norway; 32 Department of Internal Medicine and Gerontology, Jagiellonian University Medical College, Kraków, Poland; 33 Department of Geriatric Medicine, Skåne University Hospital, Malmö, Sweden; 34 Hellenic Society for the Study and Research of Ageing, Athens, Greece; 35 Laboratory for Research On Aging Society, Chair of Epidemiology and Preventive Medicine, Medical Faculty, Jagiellonian University Medical College, Kraków, Poland; 36 Department of Internal Medicine and Geriatrics, University Hospital, Kraków, Poland; 37 Department of Geriatrics and Internal Medicine, 1st Faculty of Medicine, Charles University and General University Hospital, Prague, Czech Republic; 38 Faculty of Health and Social Sciences, University of South Bohemia, Ceske Budejovice, Czech Republic; 39 Center for Geriatric Medicine, University Medical Centre Ljubljana, Ljubljana, Slovenia; 40 Faculty of Medicine and Life Sciences, Research Group Healthcare & Ethics, UHasselt, Diepenbeek, Belgium; 41 KU Leuven, Faculty of Medicine, Department of Public Health and Primary Care, Academic Centre for Nursing and Midwifery, Leuven, Belgium; 42 Faculty of Medicine, Tirana University of Medicine, Tirana, Albania

**Keywords:** Falls prevention, Implementation, Injury, Geriatric medicine, Survey

## Abstract

**Purpose:**

To explore the challenges and opportunities for the implementation of falls preventive services across Europe.

**Methods:**

An online cross-sectional survey among healthcare professionals was initiated by the European Geriatric Medicine Society (EuGMS) Special Interest Group on Falls and Fractures containing a Likert scale and multiple-choice questions on education and knowledge, current practices, barriers, and facilitators for falls prevention. Survey participation for healthcare professionals was encouraged by the EuGMS through an email invitation, website banner, and social media. National representatives from 24 countries further promoted it via societies, local networks, and hospital channels.

**Results:**

A total of 1669 multidisciplinary healthcare professionals participated from 34 European countries (median 47 years; 75% female; 40.6% physicians (73.3% geriatricians/trainees), 36% physiotherapists, 23.4% other healthcare professionals). Only 26.9% believed their undergraduate education adequately prepared them for clinical practice in this area. A total of 75.8% of respondents reported opportunistically screening older adults for fall risk often or always during consultations. Gait and balance assessment was considered the most important and was the most frequently performed component of the multifactorial fall risk assessment. The top-five barriers were staffing issues, lack of time, older adults' non-adherence to recommended strategies, workload related to falls prevention, and prioritizing other tasks. The top-five facilitators were more time, easy-to-use guidelines, sufficient resources, increased education and training on falls prevention, and increased collaboration. We observed regional and country-level variation in these top barriers and facilitators.

**Conclusion:**

This survey highlights the need for improved undergraduate education in falls prevention across Europe. It is essential to educate and engage governmental bodies and insurers to secure their support and prioritization of falls prevention initiatives. Furthermore, enhancing education, addressing older adults' nonadherence, interdisciplinary collaboration and providing easy-to-use guidelines seem crucial for effective implementation. The falls prevention strategy should be tailored to the local context.

**Supplementary Information:**

The online version contains supplementary material available at 10.1007/s41999-025-01237-5.

## Key summary points

**Aim:**

To explore education and knowledge, current practices, barriers, and facilitators for the implementation of falls prevention services among healthcare professionals in Europe.

**Findings:**

The top five barriers to implementation of falls prevention included staffing issues, time constraints, non-adherence among older adults, workload pressures related to falls prevention, and competing priorities; and the top five facilitators included more time, user-friendly guidelines, adequate resources, enhanced education and training opportunities for professionals, and strengthened inter-professional collaborative practices. There was variation between regions and countries in the ranking of top barriers and facilitators.

**Message:**

It is necessary to tailor the falls prevention implementation strategy to the local context and actively involve all stakeholders.

**Supplementary Information:**

The online version contains supplementary material available at 10.1007/s41999-025-01237-5.

## Introduction

Falls are a significant public health concern both globally and in Europe [1]. According to the World Health Organization, falls result in approximately 684,000 fatal injuries each year, making them the second leading cause of accidental or unintentional injury deaths worldwide [1]. In addition, 172 million people who have experienced a fall are left with short- or long-term disability globally each year [1]. This burden is particularly pronounced among older adults, for whom falls are the primary cause of injury-related mortality and have negative effects on functional independence and quality of life [2–4]. Furthermore, falls are one of the main modifiable causes of emergency department, hospital, and nursing home admissions [5]. Aside from the human toll, the economic impact of falls is substantial. It is estimated that up to 1.5% of total healthcare expenditure is attributed to fall-related medical care costs [6].

The first World Guidelines for Falls Prevention and Management for Older Adults (2022) (WFG) aimed to enhance the effective delivery of falls prevention [7]. They provide globally agreed-upon and evidence-based recommendations for the person-centered approach to falls prevention while facilitating knowledge dissemination. The WFG state that multidomain interventions (i.e., a combination of interventions tailored to address different risk factors) when delivered, can prevent future falls. However, the delivery of effective and accessible falls prevention itself remains challenging within current healthcare systems in Europe and worldwide. Recent large, pragmatic trials on multidomain falls prevention interventions have demonstrated no significant effect on falls outcomes [8, 9]. Challenges in adoption, fidelity, and adherence to the interventions are likely a key factor in explaining why no effect was observed in these pragmatic trials, stressing the importance of ensuring real-world falls prevention implementation.

The aim of this study was to explore the challenges and opportunities for implementation of falls preventive services across Europe among health care professionals, to help the implementation of the WFG recommendations on a European level and to inform the European Geriatric Medicine Societies’ (EuGMS) special interest group (SIG) on Falls and Fractures to formulate appropriate recommendations.

## Methods

### Design

The EuGMS SIG on Falls and Fractures initiated a European survey on the status and prospects of effective implementation of falls prevention in Europe. The cross-sectional online survey among health care professionals was facilitated by LimeSurvey. The data collection period was from December 2022 to March 2023.

### Procedure

First, a steering committee was established including experts in the field of falls prevention and communication science (LS, CB, HB, JF, R-A K, AL, JR, DS, and NvdV). The steering committee decided on the original questionnaire domains) based on expert knowledge and a preliminary review. These included: (1) participants’ characteristics, (2) knowledge and education on falls prevention, (3) falls prevention approaches and practices, and (4) barriers and facilitators for the implementation of falls prevention. Subsequently, the literature on these domains e.g., determinants of the implementation of falls prevention, was summarized, and the survey questions were drafted. The drafted survey questions in English were checked by native English speakers from the steering committee.

The original questionnaire was piloted by the steering committee members and three independent healthcare professionals to evaluate its clarity, feasibility, and the time required for completion. Based on the suggestions from the piloting panel, the questionnaire was adjusted.

The questionnaire was translated into Albanian, Czech, Finnish, French, German, Hebrew, Italian, Norwegian, Polish, Spanish, and Turkish by the national representatives whereas the English survey was used in the remainder of the countries.

Survey participation was encouraged by the EuGMS through an invitational email sent to all its members, a banner on its official website, and via social media channels. In addition, national representatives (SIG falls & fractures members) from Albania, Austria, Belgium, Czech Republic, Denmark, Finland, France, Germany, Greece, Iceland, Ireland, Israel, Italy, Malta, Netherlands, North Macedonia, Norway, Poland, Slovenia, Spain, Sweden, Türkiye, United Kingdom, who were collaborating on the project, promoted participation among their national colleagues by distributing the survey in their country through national societies, stakeholders lists, personal networks, social media, newsletters, and/or at the hospital of employment.

### Study population

Any European healthcare professional could participate in the survey. We particularly encouraged the national representatives to connect with geriatrics and physiotherapy societies, given their significant role in falls prevention. We aimed to include participants from as many European countries as possible, with a target of approximately 1000 participants based on a prior EuGMS survey project [10].

### Data Collection and questionnaire

The complete survey questionnaire is provided as Supplementary Data 1. The participants’ characteristics domain contained questions on gender, age, residing country, profession, work experience, and the setting in which the respondent is working. The knowledge and education domain included knowledge on falls prevention (Likert scale), whether undergraduate education adequately prepared for clinical practice (Likert scale), if the respondent received education and training (yes/no) and type (multiple choice), and how many hours. The falls prevention domain contained questions on screening of fall risk (Likert scale), rating the importance and frequency of carrying out fall risk assessments and interventions (Likert scale), involvement of different healthcare professionals (Likert scale), frequency of shared decision-making (Likert scale), and strategies to increase adherence to planned interventions (Likert scale). Assessment of barriers and facilitators for falls prevention included questions on barriers (Likert scale) and facilitators for the implementation of falls prevention (multiple choice).

### European regions

European regions were categorized according to the geographical definition of the United Nations (based on homogeneity in economic or social factors; https://unstats.un.org/unsd/methodology/m49/) to Western, Southern, Northern, and Eastern Europe. Turkiye and Israel were categorized as Eastern Europe based on their geographical location and in line with previous surveys [10].

### Statistical analysis

Participants’ characteristics (age, gender, residing country, profession, work experience, and setting in which the respondent is working) were analyzed for individuals who responded to at least one question in the second questionnaire domain, knowledge and education on falls prevention. We calculated frequencies and percentages for categorical variables and medians and interquartile ranges for continuous variables. For the remaining survey items, we calculated distributions of Likert scales and frequencies. Furthermore, the findings were reported per European region, and additionally, the results for countries with a minimum of 50 participants were presented individually. Additionally, the results are reported separately for physicians, physiotherapists, and other healthcare professionals grouped into one category. Furthermore, the results for current practice are reported separately across various work settings.

All statistical analyses were performed with IBM SPSS Statistics for Windows, Version 28 (IBM Corp, NY).

### Ethics

The Medical Ethics Research Committee of Amsterdam UMC declared that the Medical Research Involving Human Subjects Act did not apply to this study. In the UK, the study was approved by Newcastle University’s Faculty of Medical Sciences’ Ethics Committee (26860/2022). The study adhered to the Icelandic Act no. 90/2018 on Data Protection and the Processing of Personal Data, as outlined by the Icelandic Data Protection Authority. The Icelandic National Institutional Review Board waived the need for study approval, as the collected information was not considered personal, and all surveys were completed anonymously. Additional ethical approval was not deemed necessary for the other countries involved, given the nature of the study and in accordance with local regulations.

Prior to entering the survey questions, all participants gave digital informed consent. The participants answered the questions anonymously. Participation was voluntary and participants could withdraw at any time without any consequences.

## Results

### Participant characteristics

A total of 1669 healthcare professionals participated (Table 1). Median age of the participants was 47 years (IQR 37–56) and 75% were female (Table 1). Among participants, 40.6% were physicians, 36% physiotherapists, and 23.4% other healthcare professionals. Most physician respondents were practicing geriatricians or specialists in the care of older adults (59.9%), 10.6% were trainee geriatricians and 2.8% were non-practicing geriatricians. General practitioners accounted for 16.5% of the physicians, 1.2% were GPs in training, and 8.9% worked in other roles. The median working years’ experience as a professional healthcare worker was 20 (IQR 10–30). The survey participants were distributed across various work settings, with 29.1% working in general practice or community settings, 27.4% in hospital clinical wards, 21.8% in long-term care facilities or rehabilitation settings, 11.0% in hospital outpatient clinics, and 10.7% in other settings. Respondents worked in 34 countries.

**Table 1:** Characteristics of participants

	Total (*n* = 1669)
Age (median, IQR) (*n* = 1645)	47 (37–56)
Gender (female) (*n* = 1662)	75.0% (*n* = 1246)
Profession (*n* = 1665)	
Physician	40.6% (*n* = 676)
Physiotherapist	36.0% (*n* = 599)
Nursing professionals across all levels	11.5% (*n* = 191)
Occupational therapist	6.3% (*n* = 105)
Other	5.6% (*n* = 94)
Type of physician (*n* = 671)	
Practicing Geriatrician or specialist in care of older adults	59.9% (*n* = 402)
Trainee Geriatrician or specialist in care of older adults	10.6% (*n* = 71)
Non-practicing Geriatrician or specialist in care of older adults	2.8% (*n* = 19)
GP	16.5% (*n* = 111)
GP in training	1.2% (*n* = 8)
Other	8.9% (*n* = 60)
Healthcare working experience years (median, IQR) (*n* = 1624)	20 (10–30)
Current work environment (*n* = 1645)	
General practice or community setting	29.1% (*n* = 478)
Hospital, mostly clinical ward	27.4% (*n* = 451)
Long-term care facility or rehabilitation setting	21.8% (*n* = 359)
Hospital, mostly outpatient clinic	11.0% (*n* = 181)
Other	10.7% (*n* = 176)
Country	
Israel	*n* = 448
United Kingdom	*n* = 224
Austria	*n* = 120
Slovenia	*n* = 103
Spain	*n* = 76
Malta	*n* = 72
Sweden	*n* = 61
North Macedonia	*n* = 47
Finland	*n* = 43
Netherlands	*n* = 43
Ireland	*n* = 42
Iceland	*n* = 40
Denmark	*n* = 39
Türkiye	*n* = 37
France	*n* = 36
Czech Republic	*n* = 35
Estonia	*n* = 23
Greece	*n* = 23
Germany	*n* = 21
Poland	*n* = 21
Italy	*n* = 19
Portugal	*n* = 19
Albania	*n* = 16
Belgium	*n* = 15
Romania	*n* = 10
Norway	*n* = 9
Russia	*n* = 7
Switzerland	*n* = 7
Lithuania	*n* = 6
Belarus	*n* = 2
Andorra	*n* = 1
Bulgaria	*n* = 1
Hungary	*n* = 1
Serbia	*n* = 1
Ukraine	*n* = 1

The participant characteristics throughout the different European regions and countries are reported in the appendix (Supplementary Table 1 and Supplementary Table 2). In the western region (*n* = 242), the most represented professionals among the respondents were physicians (45%), followed by physiotherapists (22.3%), occupational therapist (17.8%), other healthcare professionals (11.9%), and nursing professionals (2.9%). In this region, the most common working environment was a long-term care facility or rehabilitation setting (40.5%). In the northern region (*n* = 487), physiotherapists (48.5%) were the most common professionals among respondents, followed by physicians (33.7%), nursing professionals (8.2%), occupational therapists (6.0%), and other healthcare professionals (3.7%). The predominant setting was hospital, mostly clinical ward (40.9%). For the eastern region (*n* = 563), physiotherapists (44.5%) represented the largest professional group, followed by physicians (37.7%), nursing professionals (10.0%), occupational therapists (5.2%), and other healthcare professionals (2.5%). Respondents most frequently worked in a general practice or community setting (44.3%). In the southern region (*n* = 377), physicians (50.9%) had the highest representation, followed by nursing professionals (23.3%), physiotherapists (15.9%), other healthcare professionals (8.8%), and occupational therapists (1.1%). The most common work setting was the hospital, mostly clinical wards (29.7%).

### Knowledge and education on falls prevention

When asked to rate their level of knowledge about falls prevention in older adults (*n* = 1663 respondents i.e. who answered this question), 55.0% reported "Very Knowledgeable," 40.5% "Some Knowledge," 4.3% "Little Knowledge," and 0.3% reported "No Knowledge." In response to the statement (*n* = 1654 respondents), "Education during my undergraduate studies prepared me adequately for falls prevention in clinical practice," 26.9% of participants agreed or strongly agreed. Data on knowledge and education in different regions and countries of Europe are reported in Appendix (Supplementary Table 3 and Supplementary Table 4). Both self-reported knowledge and satisfaction with undergraduate education varied between regions and countries. The percentage of participants who ranked themselves as very knowledgeable in falls prevention ranged from 38.2% (southern region) to 65.9% (northern region). The percentage of participants who agreed or strongly agreed that their undergraduate education adequately prepared them for clinical practice in fall prevention ranged from 17.5% (northern region) to 34.2% (eastern region). Data on knowledge and education among different health care professionals are reported in Appendix (Supplementary Table 5). The percentage of participants who agreed or strongly agreed that their undergraduate education adequately prepared them for clinical practice in fall prevention varied from 17.2% (physicians) to 35.4% (physiotherapists). A total of 30.8% of the other healthcare professionals agreed or strongly agreed. From the geriatricians/specialists in care of older adults 19.1% agreed or strongly agreed and from the trainee geriatricians 18.5% agreed or strongly agreed.

Regarding education or training on falls prevention (*n* = 1654 respondents), 81.9% of respondents indicated they had received it. Among those who received education or training on falls prevention, the cited sources, ranked based on how often they were selected, was professional development (71.0%), conferences, workshops, and other scientific meetings (69.6%), self-learning or online resources (62.3%), postgraduate studies (40.8%), undergraduate studies (25.2%), and other sources (7.3%). Participants estimated the number of hours of education or training on falls prevention they received in the last 5 years (*n* = 1568 respondents), with 36.6% reporting less than 5 h, 33.2% reporting 5 to 15 h, and 30.2% reporting more than 15 h.

### Falls prevention approaches and practices

A total of 75.8% of respondents (948/1251 respondents) reported opportunistic screening of older adults for fall risk often or always during consultations. The following components were taken into account often or always: 87.9% considered the history of falls in the past year, 87.8% assessed whether the patient felt unsteady when standing or walking, and 81.9% evaluated whether the patient expressed worry about falling. Data on falls prevention approaches and practices in different regions and countries of Europe, among different health care professionals, and across various work environments are reported in Appendix (Supplementary Tables 6, Supplementary Table 7, Supplementary Table 8 and Supplementary Table 9). Opportunistic screening, with percentages representing those who reported often/always conducting case finding, ranged from 66.8% (southern region) to 88.3% (northern region), among different health care professionals from 68.0% (other health care professionals) to 80.5% (physiotherapists) and across various work environments from 70.1% (other work environment) to 82.3% (hospital, mostly outpatient clinic).

When patients reported falls in the past year, 90.1% assessed often/always whether the patient was injured, 92.3% evaluated often/always if the patient had multiple falls, 82.0% checked often/always if the patient was unable to get up after falling, 82.9% assessed often/always if the fall was accompanied by suspected loss of consciousness, and 78.0% considered frailty often/always.

**Fig. 1:**
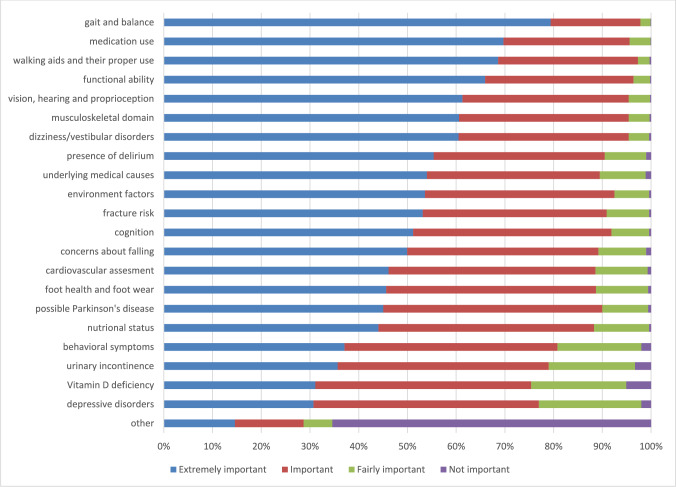
Perceived importance of assessing relevant components in a multifactorial falls risk assessment. *N* = 897 to *N* = 871 respondents for the different items except for other *n* = 885

**Fig. 2:**
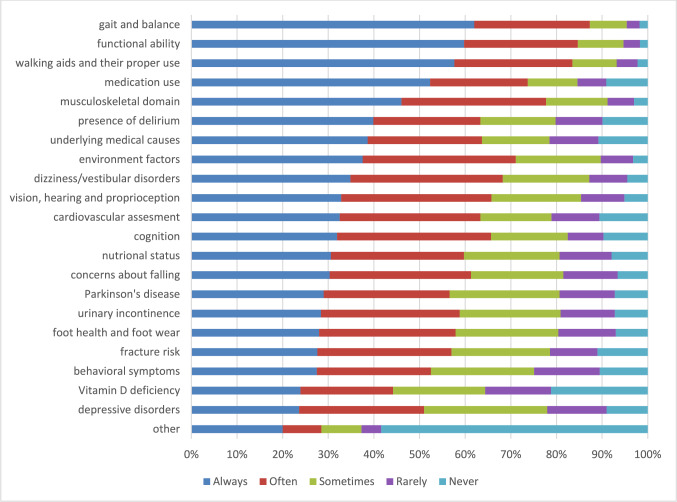
Frequency of assessing relevant components in multifactorial falls risk assessment. *N* = 1138 to *N* = 1122 respondents for the different items except for other *n* = 861

Figure 1 shows the perceived importance of different components in a multifactorial falls risk assessment and Fig. 2 shows how often the different components are performed. Gait and balance assessment was considered the most important and also the most frequently performed.

The most frequent (always/often) professionals to be involved in fall risk assessment or delivery of the intervention (81.5%, 966/1185 respondents) were physiotherapists. A total of 59.8% of respondents reported involvement of geriatricians often/always, 42.0% of general practitioners often/always, 64.8% of nurses often/always, and 55.3% of occupational therapists often/always in fall risk assessment or intervention. Dieticians (21.1% often/always), pharmacists (21.2% often/always), and other professionals (13.6% often/always) were less commonly involved.

A total of 73.6% of respondents reported often/always inquiring about older adults' perceptions of falls, their causes, future risk, and how they can be prevented, as a part of comprehensive falls assessment (867/1177 respondents). A total of 66.8% reported often/always helping the patient to explore and compare treatment options, and 82.4% considered often/always the patient’s or caregiver’s preferences and goals when developing care plans. A total of 80.1% reported often/always reaching collective decisions with patients.

A total of 58.5% of respondents (685/1171 respondents) reported using motivational interview techniques often/always to increase adherence to planned interventions, 39.9% shared patient materials often/always, 49.0% organized follow-up visits often/always, and 14.0% used other techniques often/always to increase adherence.

### Barriers and facilitators for falls prevention

**Fig. 3:**
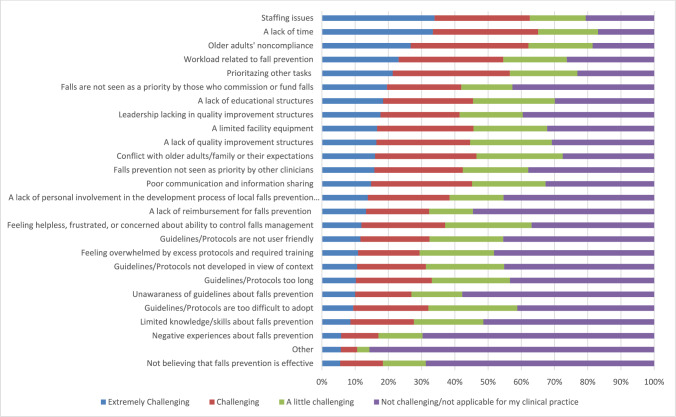
Experienced barriers for falls prevention during the past month. *N* = 897 to *N* = 871 respondents for the different items except for other *n* = 698

Figure 3 shows the barriers experienced in implementing fall prevention measures over the past month, based on how challenging they were considered. The top five barriers were staffing issues, a lack of time, older adults' nonadherence, workload related to falls prevention, and prioritizing other tasks.

Data on barriers in different regions and countries of Europe and among different health care professionals are reported in Appendix (Supplementary Table 10, Supplementary Table 11 and Supplementary Table 12). Staffing issues, a lack of time, and older adults’ nonadherence were among the top five barriers identified in each region. Conflict with older adults or their families regarding expectations ranked among the top five barriers in the eastern region. In the southern region, a lack of personal involvement in local falls prevention efforts was identified as one of the top-five barriers. Additionally, greater variation in the top barriers was observed at the country level.

**Fig. 4:**
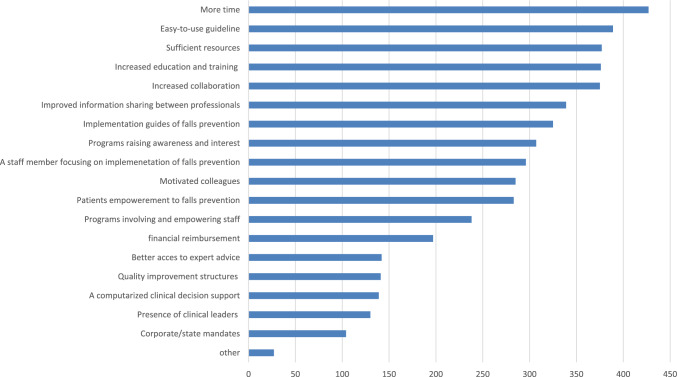
Factors facilitating further falls prevention activities. The *X*-axis indicates the number of participants selecting each factor

Figure 4 illustrates the factors that support falls prevention activities, ranked by the number of participants selecting each factor. The top five most frequently selected factors were more time, easy-to-use guidelines, sufficient resources, increased education and training, and increased collaboration.

Data on facilitators in different regions and countries of Europe and among different health care professionals are reported in Appendix (Supplementary Table 13, Supplementary Table 14 and Supplementary Table 15). More time and increased collaboration were among the top five facilitators identified in each region. Improved information sharing between professionals ranked among the top five facilitators in the northern region. Programs raising awareness and interest were identified as a top five facilitator in the eastern region. Implementation guides of falls prevention were among the top five facilitators in eastern and southern regions. As in the case of barriers, greater variation in the top facilitators was observed at the country level.

## Discussion

This European survey by the EuGMS SIG on Falls and Fractures investigated education and knowledge on falls prevention, current practices, barriers, and facilitators for implementing falls prevention among 1669 health care professionals across 34 countries. Most of the respondents were individuals who are regularly involved in falls prevention, primarily physiotherapists and geriatricians (or geriatrician trainees). The survey revealed that while most participants felt knowledgeable about falls prevention in older adults, only one quarter felt that their undergraduate education adequately prepared them for clinical practice in this area. Around two-third of the respondents reported opportunistically screening older adults for fall risk regularly during consultations across various work settings. Gait and balance assessment emerged as the most important and frequently performed component of multifactorial fall risk assessment. Physiotherapists were most involved in fall risk assessments and interventions. Furthermore, our results suggest that there is potentially room for improvement in strategies to increase adherence to planned interventions, with around 60% of respondents using techniques such as motivational interviewing, approximately 40% sharing patient materials, and about 50% organizing follow-up visits. The top barriers included staffing issues, time constraints, nonadherence among older adults, workload pressures related to falls prevention, and competing priorities. Conversely, facilitating factors included more time, user-friendly guidelines, adequate resources, enhanced education and training opportunities, and strengthened interdisciplinary collaboration. We observed regional and, even more so, country-level variation in these top barriers and facilitators.

The most frequently indicated determinants of falls prevention were resource-related health care professionals. These barriers not only limit the availability of the falls prevention services but may also impact the fidelity of intervention delivery, making it difficult for healthcare providers to consistently follow established guidelines/protocols. Our results are in line with a recent systematic review by Vandervelde et al., in which the availability of necessary resources was the most reported determinant [11]. Sufficient time, manpower, and support from relevant medical disciplines, healthcare insurers, and governmental bodies are essential for the successful implementation of falls prevention services. It is essential to educate and engage governmental bodies and insurers to secure their support and prioritization of falls prevention initiatives. However, securing additional funding and resources can be challenging, and clinicians themselves may not have direct influence over resource allocation. Therefore, it is extremely important to focus on addressing non-resource-related barriers. Many non-resource related determinants were frequently reported in our survey, including enhanced education and training opportunities, addressing older adult’s non-adherence for falls prevention strategies, user-friendly guidelines, and strengthened interdisciplinary collaboration. However, the top five barriers and facilitators differed between the countries and regions, highlighting the necessity to understand the local determinants influencing the implementation and tailoring the strategy to the local context. Recently, another systematic review by Vandervelde et al., explored implementation strategies for multifactorial falls prevention interventions in community-dwelling older persons. This review revealed that most studies concentrated on implementation strategies targeting older individuals and healthcare professionals, highlighting the significance of "tailoring," "raising awareness," and "encouraging participation". In contrast, studies focusing on strategies at the organizational, community, and policy levels emphasized the importance of "providing technical support," "engaging stakeholders," and "building coalitions” [12].

Approximately a quarter of the participants felt their undergraduate education adequately prepared them for clinical practice in falls prevention. Furthermore, a quarter of those who had received education on falls prevention identified undergraduate studies as their source. Physicians, especially, were dissatisfied with their undergraduate education in falls prevention, with no clear differences observed between geriatricians and geriatrician trainees. In some European countries and professions, education on falls prevention may be more prominently included in postgraduate education, and, furthermore, some professions, such as physiotherapy, are also taught at the postgraduate level in some European countries. However, only 40% of participants who received education on falls prevention identified postgraduate studies as their source, indicating that self-reported receipt of education during both undergraduate and postgraduate levels remains low. Our findings are in line with the recent European survey on deprescribing, where satisfaction with undergraduate training is similarly tempered [10]. These results suggest a broader pattern of dissatisfaction in undergraduate education across different geriatric domains. Falls prevention is integrated into the European undergraduate curriculum in geriatric medicine, established through a 2014 Delphi process outlining essential training requirements [13]. Both self-reported knowledge, satisfaction with undergraduate education and current practice varied between regions and countries. However, these findings should be interpreted with caution as the samples differed between countries e.g., in terms of the professions represented, experience, expertise, and the working environments and the reporting was based on participants’ self-reports of their knowledge, agreement, and actions, rather than objective measures of what is truly occurring. Further research is essential to comprehensively assess the current status of entry-level education of health care professionals as well as post-professional education in Europe. This investigation would provide valuable insights into the consistency, depth, and effectiveness of falls prevention training. Mapping the educational approaches and resources allocated to falls prevention at different institutions can highlight disparities and best practices, facilitating the development of standardized and enhanced educational frameworks for falls prevention, also on an interprofessional educational level. Furthermore, the PROGRAMMING (PROmoting GeRiAtric Medicine in countries where it is still eMergING) COST Action is actively working to identify professionals' educational needs, including those related to falls prevention, to target them more effectively and efficiently [14].

Based on the survey results and guidelines, falls prevention is inherently multidisciplinary, with various healthcare professionals playing key roles in assessing risk and implementing interventions. Most commonly involved (often or always) were physiotherapists (81.5% often/always), followed by nurses (64.8%), geriatricians (59.8%), occupational therapists (55.3%), general practitioners (42.0%), pharmacists (21.2%), and dieticians (21.1%). An interdisciplinary approach ensures a comprehensive strategy, addressing the multifaceted nature of falls prevention and leveraging the unique expertise of each discipline to optimize patient outcomes. However, the strategy of this approach can vary significantly depending on local contexts, influenced by factors such as healthcare infrastructure and available resources. These variations should be taken into account when developing local falls prevention care pathways to ensure that strategies are tailored to the specific needs of the population served.

Adherence, the extent to which a person’s behavior corresponds with agreed recommendations, is often suboptimal in falls prevention. This was demonstrated, for example, by the two recent large trials STRIDE and PreFIT, for which poor adherence to the interventions, in addition to fidelity, was listed as one of the explaining factors for the negative findings [8, 9]. According to our survey results, there is probably room for improvement in the efforts to enhance adherence to interventions Europe-wide. A total of 58.5% of the respondents reported that they use motivational interviewing techniques often/always, 39.9% shared patient materials often/always, and 49.0% organized follow-up visits often/always. Adherence among older adults to falls prevention interventions is influenced by intrinsic factors (e.g., demographics, individual factors, health factors) and extrinsic factors (e.g., caregiver support, medication factors, healthcare system, and environment) according to a recent systematic review [15]. Higher adherence was linked to factors such as higher socioeconomic status, health literacy, marriage, lower healthcare costs, effective communication, and useful policy interventions [15]. The WFG introduced a new fall risk stratification algorithm for community-dwelling older adults, which helps to distinguish high-risk individuals from those at low and intermediate risk [7]. Older adults categorized as low risk for falls should be provided with education on fall prevention and general health exercises [7]. Information should be delivered in a way that effectively influences behavior. Incorporating behavior change techniques into physical activity (PA) interventions can help reinforce shifts in attitudes and behaviors. A recent review identified seven key components of interventions that significantly improve PA levels, including goal setting, personalized feedback, and both on-site and post-intervention support [16]. For individuals at intermediate risk for falls, the same educational resources should be provided, along with targeted exercise recommendations or a referral to a physiotherapist, trained exercise instructor, or clinical exercise physiologist to improve balance, increase muscle strength, and ultimately reduce the risk of falling. Hughes et al. discussed that, in terms of exercise therapy for falls prevention, program factors such as location, duration of exercise sessions, type, frequency, intensity, and the level of supervision or contact appear to be important for adherence behavior [17]. It is desirable that multiple fall-prevention exercise interventions are made available at the local level, ensuring that older adults have access to interventions that align with their individual preferences and needs. The WFG recommends a multifactorial fall risk assessment for community-dwelling older adults at high risk to implement individualized interventions. Improving adherence to multifactorial fall prevention interventions requires a comprehensive approach that combines personalized care and tailoring interventions to individuals preferences, mentioned behavior change techniques, patient education, follow-up visits, and collaboration between healthcare professionals and the patient's support network [15].

Approximately two-thirds of respondents reported regularly screening older adults for fall risk opportunistically during consultations across various healthcare settings. Specifically, 71.0% of respondents in general practice or community settings, 81.3% in hospital clinical wards, 73.6% in long-term care facilities or rehabilitation settings, 82.3% in hospital outpatient clinics, and 70.1% in other settings reported conducting such screenings. However, according to the WFG, all older adults residing in care homes or hospital settings should be considered at high risk of falling and, therefore, should receive a multifactorial risk assessment without the need for prior screening or selection. There is considerable variation in the implementation of these guidelines across Europe, and the findings may suggest that the WFG recommendations have not yet been fully adopted. There is a clear need for greater awareness of this recommendation to omit screening of older adults and to initiate the multifactorial assessment in these settings. Opportunistic screening in these settings may, however, still occur due to limited time and staffing resources for comprehensive falls prevention activities, serving as a pragmatic approach to prioritize patients for intervention. Further research is warranted to better understand these practical constraints and to identify potential support measures that could facilitate the implementation of guideline-concordant approaches.

A key strength of this study is its pan-European scope, featuring over 1600 health care professionals from various disciplines and healthcare services. However, several limitations should be considered when interpreting the findings of this study. Firstly, the self-reported nature of responses may introduce a bias, as health care professionals may overestimate their adherence to falls prevention practices compared to their actual clinical behavior. Moreover, the survey respondents represent a sample that is inherently more interested or engaged in falls prevention and primarily physiotherapists or geriatricians. This skews the results towards more positive attitudes and practices than those observed in the broader healthcare community. Additionally, the distribution of participants across Europe was uneven, with certain regions potentially overrepresented, limiting the generalizability of findings. The questionnaire's length and complexity may have contributed to respondent fatigue, leading to dropout, impacting the comprehensiveness of the data collected. Lastly, we did not ascertain information regarding access to or availability of dedicated facilities for falls prevention.

## Conclusion

This European survey by the EuGMS SIG on Falls and Fractures revealed key insights regarding current falls prevention activities and implementation determinants. These findings can help inform and support the implementation of state-of-the-art falls prevention practices across Europe, as recommended by the WFG. The survey showed a significant gap between perceived knowledge and educational needs and preparedness from undergraduate education. Falls prevention should adopt a multidisciplinary approach to ensure comprehensive multifactorial risk assessments and effective multidomain intervention delivery, as these are central components of the WFG. Addressing resource-related barriers were prioritized as crucial for effective and falls prevention services implementation and maintenance. It is essential to educate and engage governmental bodies and insurers to secure their support and prioritization of falls prevention initiatives. This can be achieved by presenting compelling, evidence-based data, such as insights from implementation studies and big data. There is a critical need for a coordinated effort to collect and analyze data across Europe. Additionally, enhancing education, addressing older adults’ nonadherence, strengthening interdisciplinary collaboration, and providing user-friendly guidelines were found to be vital components for implementation. We identified variations between countries and regions, indicating that customizing strategies to fit local contexts will optimize the implementation of WFG-based falls prevention services.

## Supplementary Information

Below is the link to the electronic supplementary material.

Supplementary file1 (DOCX 232 KB)

Supplementary file1 (DOCX 79 KB)

## Data Availability

The data used in this study are available upon request. Interested researchers may contact the corresponding author. Access to the data will be provided after approval of the authors.

## References

[1] World Health Organization (2021) Step safely: strategies for preventing and managing falls across the life-course. World Health Organization. https://apps.who.int/iris/handle/10665/340962. License: CC BY-NC-SA 3.0 IGO

[2] EuroSafe (2016) EuroSafe. Injuries in the European Union, Summary on injury statistics 2012–2014 Amsterdam 2016. http://www.bridge-health.eu/sites/default/files/EuropeSafe_Master_R4_SinglePage_12102016%20%281%29.pdf

[3] Stel VS, Smit JH, Pluijm SM, Lips P (2004) Consequences of falling in older men and women and risk factors for health service use and functional decline. Age Ageing 33(1):58–65 14695865 10.1093/ageing/afh028

[4] Hartholt KA, van Beeck EF, Polinder S, van der Velde N, van Lieshout EM, Panneman MJ et al (2011) Societal consequences of falls in the older population: injuries, healthcare costs, and long-term reduced quality of life. J Trauma 71(3):748–753 21045738 10.1097/TA.0b013e3181f6f5e5

[5] Blain H, Miot S, Bernard PL (2021) How can we prevent falls? In: Falaschi P, Marsh D (eds) Orthogeriatrics: the management of older patients with fragility fractures. Springer, Cham, pp 273–290 33347100

[6] Heinrich S, Rapp K, Rissmann U, Becker C, König H-H (2010) Cost of falls in old age: a systematic review. Osteoporos Int 21(6):891–902 19924496 10.1007/s00198-009-1100-1

[7] Montero-Odasso M, van der Velde N, Martin FC, Petrovic M, Tan MP, Ryg J et al (2022) World guidelines for falls prevention and management for older adults: a global initiative. Age Ageing 51(9):afac205 36178003 10.1093/ageing/afac205 PMC9523684

[8] Bhasin S, Gill TM, Reuben DB, Latham NK, Ganz DA, Greene EJ et al (2020) A randomized trial of a multifactorial strategy to prevent serious fall injuries. N Engl J Med 383(2):129–140 32640131 10.1056/NEJMoa2002183 PMC7421468

[9] Bruce J, Hossain A, Lall R, Withers EJ, Finnegan S, Underwood M et al (2021) Fall prevention interventions in primary care to reduce fractures and falls in people aged 70 years and over: the PreFIT three-arm cluster RCT. Health Technol Assess 25(34):1–114 34075875 10.3310/hta25340 PMC8200932

[10] van Poelgeest EP, Seppala LJ, Lee JM, Bahat G, Ilhan B, Lavan AH et al (2022) Deprescribing practices, habits and attitudes of geriatricians and geriatricians-in-training across Europe: a large web-based survey. Eur Geriatr Med 13(6):1455–1466 36319837 10.1007/s41999-022-00702-9 PMC9722796

[11] Vandervelde S, Van den Bosch N, Vlaeyen E, Dierckx de Casterlé B, Flamaing J, Belaen G et al (2024) Determinants influencing the implementation of multifactorial falls risk assessment and multidomain interventions in community-dwelling older people: a systematic review. Age Ageing. 10.1093/ageing/afae123 38952187 10.1093/ageing/afae123

[12] Vandervelde S, Vlaeyen E, de Casterlé BD, Flamaing J, Valy S, Meurrens J et al (2023) Strategies to implement multifactorial falls prevention interventions in community-dwelling older persons: a systematic review. Implement Sci 18(1):4 36747293 10.1186/s13012-022-01257-w PMC9901093

[13] Masud T, Blundell A, Gordon AL, Mulpeter K, Roller R, Singler K et al (2014) European undergraduate curriculum in geriatric medicine developed using an international modified Delphi technique. Age Ageing 43(5):695–702 24603283 10.1093/ageing/afu019 PMC4143490

[14] Savas S, Demiral Yilmaz N, Kotsani M, Piotrowicz K, Duque S (2024) Which stakeholders should be addressed to promote Geriatric Medicine among healthcare professionals, educationalists and policy-makers in European countries? The PROGRAMMING COST 21,122 action experience. Aging Clin Exp Res 36(1):194 39312128 10.1007/s40520-024-02841-4 PMC11420320

[15] Santhagunam SN, Li EPH, Buschert K, Davis JC (2021) A theoretical framework to improve adherence among older adults to recommendations received at a falls prevention clinic: a narrative review. Appl Nurs Res 62:151493 34814997 10.1016/j.apnr.2021.151493

[16] Ahmed S, Lazo Green K, McGarrigle L, Money A, Pendleton N, Todd C (2024) Interventions based on behavior change techniques to encourage physical activity or decrease sedentary behavior in community-dwelling adults aged 50–70: systematic review with intervention component analysis. J Aging Phys Act 32(4):554–577 38663855 10.1123/japa.2023-0140

[17] Hughes KJ, Salmon N, Galvin R, Casey B, Clifford AM (2019) Interventions to improve adherence to exercise therapy for falls prevention in community-dwelling older adults: systematic review and meta-analysis. Age Ageing 48(2):185–195 30358800 10.1093/ageing/afy164

