# Supplementary material for: Challenges and opportunities for falls prevention: an online survey across European healthcare professionals
Source: Eur Geriatr Med. 2025 Jun 17;16(4):1269–82. doi: 10.1007/s41999-025-01237-5 (PMC12378296; doi:10.1007/s41999-025-01237-5)
Supplement: Supplementary file 1 — Supplementary file1 (DOCX 232 KB) [file 41999_2025_1237_MOESM1_ESM.pdf]

LimeSurvey

No syntax errors detected in this survey.

Logic File for Survey # [782434]: Implementation of falls prevention: a European survey

Welcome:	Dear colleague,<br><br>Thank you for considering participation in this online survey.<br><br>Falls and related injuries form a growing healthcare problem in our aging societies. To tackle this healthcare issue, many countries have developed healthcare services for falls prevention. However, they meet several challenges in the implementation of falls prevention.<br><br>The EuGMS SIG on Falls & Fractures developed this online questionnaire in an effort to capture the challenges and opportunities across Europe and identify key areas for improvement in the future. It includes questions on your current falls prevention practices, approaches, knowledge, and needs/wishes for future interventions.<br><br>This survey will take approximately 15 minutes of your time and was approved by the Medical Ethical committee of the Academic Medical Center in Amsterdam. Your personal information and data will be processed anonymously and will not be distributed to third parties. Participation is voluntary.<br><br>Yours sincerely,<br><br>On behalf of the EuGMS SIG Falls and Fractures,<br><br>Dr. Lotta Seppala and Prof. Nathalie van der Velde
End message:	Thank you for your participation, if you have any questions, please contact the researcher: Lotta Seppala, l.j.seppala@amsterdamumc.nl

#	Name [ID]	Relevance [Validation] (Default value)	Text [Help] (Tip)
G-0	**Informed consent** [GID 7009]	1	
Q-0	***Consent** [QID 106314] Multiple choice [M]	1	Before proceeding, please confirm that you have read the informed consent on the previous page and tick the box if you agree with these terms.<br><br>**Question attribute** / **Value**:<br>assessment_value: 1<br>display_columns: 1<br>statistics_showgraph: 1

SQ-1	**Consent_1**		I have read and accepted the aforementioned and I will participate in this survey.
G-1	**Part 1 participants' characteristic**<br>[GID 7010]	1	
Q-1	***Age**<br>[QID 106315]<br>Numerical input [N]	1	1. What is your age?<br><br>[Help: *Please indicate a numerical value (e.g. 40)*]<br>**Question attribute** / **Value**<br>statistics_showgraph: 1<br>max_num_value_n: 100<br>min_num_value_n: 18<br>suffix: years
Q-2	***Sex**<br>[QID 106316]<br>List (radio) [L]	1	2. What is your sex?<br>**Question attribute** / **Value**<br>display_columns: 1<br>statistics_showgraph: 1
A[0]-1	**1**	[VALUE: 0]	Male
A[0]-2	**2**	[VALUE: 0]	Female
A[0]-3	**3**	[VALUE: 0]	Other
A[0]-4	**4**	[VALUE: 0]	Prefer not to say
Q-3	***Profession**<br>[QID 106317]<br>List (radio) [L]	1	3. What is your profession?<br>**Question attribute** / **Value**<br>display_columns: 1<br>statistics_showgraph: 1
A[0]-1	**1**	[VALUE: 0]	Physician
A[0]-2	**2**	[VALUE: 0]	Physiotherapist
A[0]-3	**3**	[VALUE: 0]	Advanced Registered Nurse Practitioner
A[0]-4	**4**	[VALUE: 0]	Physician's Assistant
A[0]-5	**5**	[VALUE: 0]	Nurse
A[0]-6	**6**	[VALUE: 0]	Occupational therapist
A[0]-7	**7**	[VALUE: 0]	Other
Q-4	***ProfessionX**<br>[QID 106318]<br>Long free text [T]	((**Profession.NAOK** == "7"))	Please specify other profession<br>**Question attribute** / **Value**<br>statistics_showgraph: 1
Q-5	***ProfessPhys**<br>[QID 106319]<br>List (radio) [L]	((**Profession.NAOK** == "1"))	4. Which of the following best describes you at this moment?<br>**Question attribute** / **Value**

			display_columns: 1<br>statistics_showgraph: 1
A[0]-1	**1**	[VALUE: 0]	Practicing Geriatrician or specialist in care of older adults
A[0]-2	**2**	[VALUE: 0]	Trainee Geriatrician or specialist in care of older adults
A[0]-3	**3**	[VALUE: 0]	Non-practicing Geriatrician or specialist in care of older adults
A[0]-4	**4**	[VALUE: 0]	GP
A[0]-5	**5**	[VALUE: 0]	GP in training
A[0]-6	**6**	[VALUE: 0]	Other
Q-6	***ProfessPhysX**<br>[QID 106320]<br>Long free text [T]	((**ProfessPhys.NAOK** == "6"))	Please specify other physician type<br>Question attribute: Value<br>statistics_showgraph: 1
Q-7	***CountryJob**<br>[QID 106321]<br>List (radio) [L]	1	5. In which country are you currently working?<br>Question attribute: Value<br>display_columns: 1<br>statistics_showgraph: 1
A[0]-1	**1**	[VALUE: 0]	Albania
A[0]-2	**2**	[VALUE: 0]	Andorra
A[0]-3	**3**	[VALUE: 0]	Austria
A[0]-4	**4**	[VALUE: 0]	Belarus
A[0]-5	**5**	[VALUE: 0]	Belgium
A[0]-6	**6**	[VALUE: 0]	Bosnia
A[0]-7	**7**	[VALUE: 0]	Bulgaria
A[0]-8	**8**	[VALUE: 0]	Croatia
A[0]-9	**9**	[VALUE: 0]	Czech Republic
A[0]-10	**10**	[VALUE: 0]	Denmark
A[0]-11	**11**	[VALUE: 0]	Estonia
A[0]-12	**12**	[VALUE: 0]	Finland
A[0]-13	**13**	[VALUE: 0]	France
A[0]-14	**14**	[VALUE: 0]	Germany
A[0]-15	**15**	[VALUE: 0]	Greece
A[0]-16	**16**	[VALUE: 0]	Hungary
A[0]-17	**17**	[VALUE: 0]	Iceland
A[0]-18	**18**	[VALUE: 0]	Ireland
A[0]-19	**19**	[VALUE: 0]	Italy
A[0]-20	**20**	[VALUE: 0]	Latvia
A[0]-21	**21**	[VALUE: 0]	Liechtenstein
A[0]-22	**22**	[VALUE: 0]	Lithuania

A[0]-23	**23**	[VALUE: 0]	Luxemburg
A[0]-24	**24**	[VALUE: 0]	Malta
A[0]-25	**25**	[VALUE: 0]	Moldova
A[0]-26	**26**	[VALUE: 0]	Monaco
A[0]-27	**27**	[VALUE: 0]	Montenegro
A[0]-28	**28**	[VALUE: 0]	Netherlands
A[0]-29	**29**	[VALUE: 0]	North Macedonia
A[0]-30	**30**	[VALUE: 0]	Norway
A[0]-31	**31**	[VALUE: 0]	Poland
A[0]-32	**32**	[VALUE: 0]	Portugal
A[0]-33	**33**	[VALUE: 0]	Romania
A[0]-34	**34**	[VALUE: 0]	Russia
A[0]-35	**35**	[VALUE: 0]	San Marino
A[0]-36	**36**	[VALUE: 0]	Serbia
A[0]-37	**37**	[VALUE: 0]	Slovakia
A[0]-38	**38**	[VALUE: 0]	Slovenia
A[0]-39	**39**	[VALUE: 0]	Spain
A[0]-40	**40**	[VALUE: 0]	Sweden
A[0]-41	**41**	[VALUE: 0]	Switzerland
A[0]-42	**42**	[VALUE: 0]	Ukraine
A[0]-43	**43**	[VALUE: 0]	United Kingdom
A[0]-44	**44**	[VALUE: 0]	Vatican City
A[0]-45	**45**	[VALUE: 0]	Israel
A[0]-46	**46**	[VALUE: 0]	Turkye
A[0]-47	**47**	[VALUE: 0]	Other
Q-8	***CountryJobX**<br>[QID 106348]<br>Long free text [T]	((**CountryJob.NAOK** == "47"))	Please specify other<br><br>**Question attribute** / **Value**<br>statistics_showgraph: 1
Q-9	***ExperienceYrs**<br>[QID 106322]<br>Numerical input [N]	1	6. How many years of working experience do you have as a professional health care worker since qualified, e.g. as a medical doctor or physiotherapist?<br><br>**Question attribute** / **Value**<br>statistics_showgraph: 1<br>max_num_value_n: 60<br>min_num_value_n: 0<br>suffix: years
Q-10	***WorkEnvironm**<br>[QID 106323]<br>List (radio) [L]	1	7. Which of the following best describes your current working environment?<br><br>**Question attribute** / **Value**

			display_columns: 1<br>statistics_showgraph: 1
A[0]-1	**1**	[VALUE: 0]	General practice or community setting
A[0]-2	**2**	[VALUE: 0]	Hospital, mostly outpatient clinic
A[0]-3	**3**	[VALUE: 0]	Hospital, mostly clinical ward
A[0]-4	**4**	[VALUE: 0]	Long-term care facility (e.g. nursing home) or rehabilitation setting
A[0]-5	**5**	[VALUE: 0]	Other
Q-11	***WorkEnvironmX**<br>[QID 106324]<br>Long free text [T]	((**WorkEnvironm.NAOK** == "5"))	Please specify other working environment<br>**Question attribute** / **Value**<br>statistics_showgraph: 1
G-2	**Part 2 Knowledge and education on falls prevention**<br>[GID 7011]	**1**	
Q-12	***RatingKnowlFalls**<br>[QID 106325]<br>List (radio) [L]	**1**	8. How would you rate your level of knowledge about falls prevention in older adults?<br>**Question attribute** / **Value**<br>display_columns: 1<br>statistics_showgraph: 1
A[0]-1	**1**	[VALUE: 0]	No knowledge
A[0]-2	**2**	[VALUE: 0]	Little knowledge
A[0]-3	**3**	[VALUE: 0]	Some knowledge
A[0]-4	**4**	[VALUE: 0]	Very knowledgeable
Q-13	***EducFallsPrevent**<br>[QID 106326]<br>List (radio) [L]	**1**	9. Education during my undergraduate studies prepared me adequately for falls prevention in clinical practice.<br>**Question attribute** / **Value**<br>display_columns: 1<br>statistics_showgraph: 1
A[0]-1	**1**	[VALUE: 0]	Strongly disagree
A[0]-2	**2**	[VALUE: 0]	Disagree
A[0]-3	**3**	[VALUE: 0]	Neutral
A[0]-4	**4**	[VALUE: 0]	Agree
A[0]-5	**5**	[VALUE: 0]	Strongly agree
Q-14	***EducFPReceivYN**<br>[QID 106327]<br>List (radio) [L]	**1**	10. Have you ever received education or training sessions on falls prevention?<br>[Help: e.g. under- or post graduates studies, self-learning, online resources,

			professional development, conferences and workshops]<br><br>Question attribute / Value<br>display_columns: 1<br>statistics_showgraph: 1
A[0]-1	**1**	[VALUE: 0]	Yes
A[0]-2	**2**	[VALUE: 0]	No
Q-15	***EducFPRecType**<br>[QID 106328]<br>Multiple choice [M]	((**EducFPReceivYN.NAOK** == "1"))	11. Where did you receive this education or training on falls prevention? *(Please choose all that apply.)*<br><br>Question attribute / Value<br>assessment_value: 1<br>display_columns: 1<br>statistics_showgraph: 1
SQ-1	**EducFPRecType_1**		Undergraduate studies
SQ-2	**EducFPRecType_2**		Postgraduate studies
SQ-3	**EducFPRecType_3**		Self-learning or online resources
SQ-4	**EducFPRecType_4**		Professional development (e.g. Continuing professional development courses, in-service training)
SQ-5	**EducFPRecType_5**		Conferences, workshops and other scientific meetings
SQ-6	**EducFPRecType_6**		Other
Q-16	***EducFPRecTypeX**<br>[QID 106329]<br>Long free text [T]	((**EducFPRecType_6.NAOK** == "Y"))	Please specify other type of training<br><br>Question attribute / Value<br>statistics_showgraph: 1
Q-17	***FPTrainHrs**<br>[QID 106330]<br>List (radio) [L]	**1**	12. Please estimate the number of hours of education or training regarding falls prevention you have received in the last 5 years.<br><br>[Help:<br>estimation is sufficient if you do not know exactly<br>]<br><br>Question attribute / Value<br>display_columns: 1<br>statistics_showgraph: 1
A[0]-1	**1**	[VALUE: 0]	Less than 5 hours
A[0]-2	**2**	[VALUE: 0]	5 to 15 hours
A[0]-3	**3**	[VALUE: 0]	Over 15 hours

Q-18	***FPGuidelineYN** [QID 106331] Multiple choice [M]	1	13. Do you use existing national or international fall prevention guidelines to guide your clinical work? *(Please select all that apply.)*<br><br>**Question attribute** / **Value**<br>assessment_value: 1<br>display_columns: 1<br>exclude_all_others: 3<br>statistics_showgraph: 1
SQ-1	**FPGuidelineYN_1**	**is_empty**(**FPGuidelineYN_3.NAOK**)	Yes, national guideline
SQ-2	**FPGuidelineYN_2**	**is_empty**(**FPGuidelineYN_3.NAOK**)	Yes, international guideline
SQ-3	**FPGuidelineYN_3**		No
Q-19	***FPGuidelineX** [QID 106332] Long free text [T]	((**FPGuidelineYN_1.NAOK** == "Y")) or ((**FPGuidelineYN_2.NAOK** == "Y"))	14. Please specify its name or share the link if it is an open source document<br><br>**Question attribute** / **Value**<br>statistics_showgraph: 1
G-3	**Part 3 Your Falls Prevention Approaches and Practices** [GID 7012]	1	
Q-20	***RatingCaseFind** [QID 106333] Array [F]	1	15. Please rate each of the following statements **on** fall risk stratification for falls prevention in older patients. How often do you do the following? If you are not currently doing clinical work (e.g. you focus completely on research), answer rest of the questions as you would when you last did clinical work.<br><br>[Help: Opportunistic screening: screening patients that present to health services not due to fall e.g. during an annual health visit]<br><br>**Question attribute** / **Value**<br>answer_width: 45<br>statistics_showgraph: 1
SQ-1	**RatingCaseFind_1**		**1. During my consultations I opportunistically screen older adults for fall risk**
SQ-2	**RatingCaseFind_2**		**2. I take the following components into account when opportunistically screening for fall risk:**<br><br>a. history of falls in the past year

SQ-3	**RatingCaseFind_3**		b. whether the patient is feeling unsteady when standing or walking
SQ-4	**RatingCaseFind_4**		c. whether the patient is worried about falling
SQ-5	**RatingCaseFind_5**		d. other
SQ-6	**RatingCaseFind_6**		**3. When the patient reports that (s)he has fallen in the past year, I assess:**<br>a. whether she or he was injured
SQ-7	**RatingCaseFind_7**		b whether (s)he had multiple falls
SQ-8	**RatingCaseFind_8**		c. whether (s)he was laying on the floor and was unable to get up
SQ-9	**RatingCaseFind_9**		d. whether the fall was accompanied by (suspected) loss of consciousness
SQ-10	**RatingCaseFind_10**		e. frailty
SQ-11	**RatingCaseFind_11**		f. other
A[0]-1	**1**	[VALUE: 0]	Never
A[0]-2	**2**	[VALUE: 0]	Rarely
A[0]-3	**3**	[VALUE: 0]	Sometimes
A[0]-4	**4**	[VALUE: 0]	Often
A[0]-5	**5**	[VALUE: 0]	Always
A[0]-6	**99**	[VALUE: 0]	Not applicable
Q-21	***RatingCaseFind2X**<br>[QID 106339]<br>Long free text [T]	((**RatingCaseFind_5.NAOK** == "2")) or ((**RatingCaseFind_5.NAOK** == "3")) or ((**RatingCaseFind_5.NAOK** == "4")) or ((**RatingCaseFind_5.NAOK** == "5"))	Please name other components<br>**Question attribute** / **Value**: statistics_showgraph / 1

Q-22	*RatingCaseFind3X<br>[QID 106334]<br>Long free text [T]	((RatingCaseFind_11.NAOK == "2")) or ((RatingCaseFind_11.NAOK == "3")) or ((RatingCaseFind_11.NAOK == "4")) or ((RatingCaseFind_11.NAOK == "5"))	Please name other assessment<br>Question attribute: statistics_showgraph — Value: 1
Q-23	*RatingRiskAssInterv<br>[QID 106335]<br>Array dual scale [1]	1	16. Please rate the following statements on fall risk assessment and interventions:<br>Question attribute / Value:<br>answer_width — 45<br>repeat_headings — 11<br>dualscale_headerA — How important is it that the following assessment is carried out<br>dualscale_headerB — How often do you do the following, when you (or the multidisciplinary team in which you are included) evaluate falls risk<br>statistics_showgraph — 1
SQ-1	RatingRiskAssInterv_1		a. cardiovascular assessment
SQ-2	RatingRiskAssInterv_2		b. assessment of cognition (e.g., MMSE)
SQ-3	RatingRiskAssInterv_3		c. assessment of vision, hearing, proprioception
SQ-4	RatingRiskAssInterv_4		d. assessment of gait and balance
SQ-5	RatingRiskAssInterv_5		e. assessment of musculoskeletal domain
SQ-6	RatingRiskAssInterv_6		f. assessment of fracture risk
SQ-7	RatingRiskAssInterv_7		g. assessment of fear of falling
SQ-8	RatingRiskAssInterv_8		h. assessment of medication use (i.e., medication review)
SQ-9	RatingRiskAssInterv_9		i. assessment of Vitamin D deficiency
SQ-10	RatingRiskAssInterv_10		j. assessment for underlying medical causes (or falls as an atypical presentation of another condition e.g. infection)
SQ-11	RatingRiskAssInterv_11		k. assessment of foot health and foot wear
SQ-12	RatingRiskAssInterv_12		l. assessment of environmental factors

SQ-13	**RatingRiskAssInterv_13**		m. assessment of nutritional status
SQ-14	**RatingRiskAssInterv_14**		n. assessment of urinary incontinence
SQ-15	**RatingRiskAssInterv_15**		o. assessment presence of delirium
SQ-16	**RatingRiskAssInterv_16**		p. assessment of dizziness/vestibular disorders
SQ-17	**RatingRiskAssInterv_17**		q. assessment of depressive disorders
SQ-18	**RatingRiskAssInterv_18**		r. assessment of functional ability
SQ-19	**RatingRiskAssInterv_19**		s. assessment of possible Parkinson's disease
SQ-20	**RatingRiskAssInterv_20**		t. assessment of walking aids and their proper use
SQ-21	**RatingRiskAssInterv_21**		u. assessment of behavioral symptoms such as impulsiveness and agitation
SQ-22	**RatingRiskAssInterv_22**		v. other
A[0]-1	**1**	[VALUE: 0]	Not important
A[0]-2	**2**	[VALUE: 0]	Fairly important
A[0]-3	**3**	[VALUE: 0]	Important
A[0]-4	**4**	[VALUE: 0]	Extremely important
A[1]-1	**1**	[VALUE: 0]	Never
A[1]-2	**2**	[VALUE: 0]	Rarely
A[1]-3	**3**	[VALUE: 0]	Sometimes
A[1]-4	**4**	[VALUE: 0]	Often
A[1]-5	**5**	[VALUE: 0]	Always
Q-24	***RatingRiskAssX** [QID 106336] Long free text [T]	((**RatingRiskAssInterv_22_0.NAOK** == "2")) or ((**RatingRiskAssInterv_22_0.NAOK** == "3")) or ((**RatingRiskAssInterv_22_0.NAOK** == "4")) or ((**RatingRiskAssInterv_22_1.NAOK** == "2")) or ((**RatingRiskAssInterv_22_1.NAOK** == "3")) or ((**RatingRiskAssInterv_22_1.NAOK** == "4")) or ((**RatingRiskAssInterv_22_1.NAOK** == "5"))	Please name other risk assesment<br>**Question attribute**: statistics_showgraph — **Value**: 1
Q-25	***RatingFallPrevent** [QID 106337] Array [F]	**1**	17. Please rate the following persons on falls prevention in older patients:<br><br>In my clinical practice the following persons are involved in the fall risk assessment or in the delivery of intervention<br><br>[Help: We are not referring to an individual patient with the question, but

			we are interested in what happens to patients on average.]<br>Question attribute | Value<br>answer_width | 45<br>repeat_headings | 11<br>statistics_showgraph | 1
SQ-1	**RatingFallPrevent_1**		a. geriatricians
SQ-2	**RatingFallPrevent_2**		b. general practitioners
SQ-3	**RatingFallPrevent_3**		c. nurses
SQ-4	**RatingFallPrevent_4**		d. physiotherapists
SQ-5	**RatingFallPrevent_5**		e. occupational therapists
SQ-6	**RatingFallPrevent_6**		f. dietician
SQ-7	**RatingFallPrevent_7**		g. pharmacist
SQ-8	**RatingFallPrevent_8**		h. other
A[0]-1	**1**	[VALUE: 0]	Never
A[0]-2	**2**	[VALUE: 0]	Rarely
A[0]-3	**3**	[VALUE: 0]	Sometimes
A[0]-4	**4**	[VALUE: 0]	Often
A[0]-5	**5**	[VALUE: 0]	Always
A[0]-6	**99**	[VALUE: 0]	Not applicable
Q-26	***RatingFallAssessX**<br>[QID 106340]<br>Long free text [T]	((**RatingFallPrevent_8.NAOK** == "2")) or ((**RatingFallPrevent_8.NAOK** == "3")) or ((**RatingFallPrevent_8.NAOK** == "4")) or ((**RatingFallPrevent_8.NAOK** == "5"))	Please name others involved in risk assessment or in the delivery of intervention<br>Question attribute | Value<br>statistics_showgraph | 1
Q-27	***RatingSharDecis**<br>[QID 106338]<br>Array [F]	1	18. Please rate the following statements on shared-decision making. How often do you do the following?<br>Question attribute | Value<br>answer_width | 45<br>statistics_showgraph | 1
SQ-1	**RatingSharDecis_1**		As part of a comprehensive falls assessment, I inquire about the perceptions the older adult holds about falls, their causes, future risk, and how they can be prevented.
SQ-2	**RatingSharDecis_2**		I help the patient explore and compare falls prevention treatment options.
SQ-3	**RatingSharDecis_3**		When developing a falls prevention care plan, I take the patient/caregiver's preferences and goals into account.
SQ-4	**RatingSharDecis_4**		I reach a collective decision with a patient on falls prevention intervention.

A[0]-1	**1**	[VALUE: 0]	Never
A[0]-2	**2**	[VALUE: 0]	Rarely
A[0]-3	**3**	[VALUE: 0]	Sometimes
A[0]-4	**4**	[VALUE: 0]	Often
A[0]-5	**5**	[VALUE: 0]	Always
A[0]-6	**99**	[VALUE: 0]	Not applicable
Q-28	***RatingAdherInterv** [QID 106346] Array [F]	1	19. Please rate the following statements on adherence: How often do you do the following? To increase the adherence with the planned interventions<br>Question attribute / Value<br>answer_width: 45<br>statistics_showgraph: 1
SQ-1	**RatingAdherInterv_1**		a. I use motivational interview techniques
SQ-2	**RatingAdherInterv_2**		b. I share patient letters and folder with the patient
SQ-3	**RatingAdherInterv_3**		c. I organize follow-up visits to ensure execution of the planned interventions
SQ-4	**RatingAdherInterv_4**		d. Other, please name
A[0]-1	**1**	[VALUE: 0]	Never
A[0]-2	**2**	[VALUE: 0]	Rarely
A[0]-3	**3**	[VALUE: 0]	Sometimes
A[0]-4	**4**	[VALUE: 0]	Often
A[0]-5	**5**	[VALUE: 0]	Always
A[0]-6	**99**	[VALUE: 0]	Not applicable
Q-29	***RatingAdherIntervX** [QID 106347] Long free text [T]	((**RatingAdherInterv_4.NAOK** == "2")) **or** ((**RatingAdherInterv_4.NAOK** == "3")) **or** ((**RatingAdherInterv_4.NAOK** == "4")) **or** ((**RatingAdherInterv_4.NAOK** == "5"))	Please name other:<br>Question attribute / Value<br>statistics_showgraph: 1
G-4	**Part 4: Barriers and challenges** [GID 7013]	1	
Q-30	***FallsPrevImplem** [QID 106341] Array dual scale [1]	1	20. Please mark the following statements on falls prevention implementation<br>[Help: We are referring to implementation of falls prevention in

			general and not focusing on specific components of falls prevention.]<br><br>Question attribute / Value:<br>answer_width: 45<br>dualscale_headerA: In the past month, I found the implementation of falls prevention challenging due to...<br>dualscale_headerB: In the past month, I haven't implemented falls prevention due to...<br>statistics_showgraph: 1
SQ-1	**FallsPrevImplem_1**		A lack of reimbursement for falls prevention
SQ-2	**FallsPrevImplem_2**		A lack of personal involvement in the development process of local falls prevention program
SQ-3	**FallsPrevImplem_3**		Staffing issues
SQ-4	**FallsPrevImplem_4**		A lack of educational structures
SQ-5	**FallsPrevImplem_5**		A lack of time
SQ-6	**FallsPrevImplem_6**		A limited facility equipment
SQ-7	**FallsPrevImplem_7**		Prioritizing other tasks
SQ-8	**FallsPrevImplem_8**		A lack of quality improvement structures
SQ-9	**FallsPrevImplem_9**		Workload related to fall prevention<br><br>e.g. struggling to sustain interventions and the related burden of documentation and paperwork
SQ-10	**FallsPrevImplem_10**		Falls prevention guidelines/Protocols are too difficult to adopt
SQ-11	**FallsPrevImplem_11**		Falls prevention guidelines/Protocols are not developed in view of context
SQ-12	**FallsPrevImplem_12**		Falls prevention guidelines/Protocols are too long
SQ-13	**FallsPrevImplem_13**		Falls prevention guidelines/Protocols are not user friendly
SQ-14	**FallsPrevImplem_14**		Poor communication and information sharing
SQ-15	**FallsPrevImplem_15**		Leadership lacking in quality improvement skills (e.g., lack of

			leaders providing support and supervising implementation)
SQ-16	**FallsPrevImplem_16**		Oder adults' noncompliance (e.g. not completing a program of exercises)
SQ-17	**FallsPrevImplem_17**		Conflict with older adults/family or their expectations
SQ-18	**FallsPrevImplem_18**		Feeling helpless, frustrated, or concerned about ability to control fall management
SQ-19	**FallsPrevImplem_19**		Feeling overwhelmed by excess protocols and required training
SQ-20	**FallsPrevImplem_20**		Limited knowledge and skills about falls prevention
SQ-21	**FallsPrevImplem_21**		Not believing that falls prevention is effective
SQ-22	**FallsPrevImplem_22**		Unawareness of guidelines about falls prevention
SQ-23	**FallsPrevImplem_23**		Negative experiences about falls prevention
SQ-24	**FallsPrevImplem_24**		Falls prevention not seen as priority by other clinicians
SQ-25	**FallsPrevImplem_25**		Falls are not seen as a priority by those who commission or fund falls services in your region
SQ-26	**FallsPrevImplem_26**		Other
A[0]-1	**1**	[VALUE: 0]	Not challenging
A[0]-2	**2**	[VALUE: 0]	A little challenging
A[0]-3	**3**	[VALUE: 0]	Challenging
A[0]-4	**4**	[VALUE: 0]	Extremely challenging
A[0]-5	**99**	[VALUE: 0]	Not applicable
A[1]-1	**1**	[VALUE: 0]	Yes
A[1]-2	**2**	[VALUE: 0]	No
A[1]-3	**99**	[VALUE: 0]	Not applicable
Q-31	***FallsPrevImplemOthX** [QID 106342] Long free text [T]	((**FallsPrevImplem_26_0.NAOK** == "1" or **FallsPrevImplem_26_0.NAOK** == "2" or **FallsPrevImplem_26_0.NAOK** == "3" or **FallsPrevImplem_26_0.NAOK** == "4") or (**FallsPrevImplem_26_1.NAOK** == "1" or **FallsPrevImplem_26_1.NAOK** == "2"))	Please name other<br>Question attribute: statistics_showgraph — Value: 1
Q-32	***FactFallActiv** [QID 106343] Multiple choice [M]	1	21. Which of the following factors would most likely further facilitate your falls prevention activities? *(Please select 5 options that most apply to you.)*

			[Help: Choosing more than 5 answers will provide a pop-up stating that one or more questions have not been answered in a valid manner. Please re-check your answers.]<br><br>Question attribute / Value<br>assessment_value: 1<br>display_columns: 1<br>statistics_showgraph: 1<br>max_answers: 5<br>min_answers: 1<br>random_order: 1
SQ-1	**FactFallActiv_1**		More time
SQ-2	**FactFallActiv_2**		Patients empowerment to falls prevention
SQ-3	**FactFallActiv_3**		Implementation guides of falls prevention
SQ-4	**FactFallActiv_4**		Easy-to-use guideline
SQ-5	**FactFallActiv_5**		Programs raising awareness and interest
SQ-6	**FactFallActiv_6**		Motivated colleagues
SQ-7	**FactFallActiv_7**		Improved information sharing between different health care professionals
SQ-8	**FactFallActiv_8**		Programs involving and empowering staff
SQ-9	**FactFallActiv_9**		Presence of clinical leaders
SQ-10	**FactFallActiv_10**		Increased collaboration; possibility for collaboration with various disciplines
SQ-11	**FactFallActiv_11**		Increased education and training
SQ-12	**FactFallActiv_12**		Quality improvement structures
SQ-13	**FactFallActiv_13**		Corporate and/or state mandates
SQ-14	**FactFallActiv_14**		Sufficient resources
SQ-15	**FactFallActiv_15**		A staff member focusing on implementation of falls prevention
SQ-16	**FactFallActiv_16**		A computerized clinical decision support
SQ-17	**FactFallActiv_17**		Better access to expert advice
SQ-18	**FactFallActiv_18**		Financial reimbursement
SQ-19	**FactFallActiv_19**		Other
Q-33	***FactFallActivX**<br>[QID 106345]<br>Long free text [T]	((**FactFallActiv_19.NAOK** == "Y"))	Please specify other:<br><br>Question attribute / Value<br>statistics_showgraph: 1
G-5	**Final message**<br>[GID 7014]	1	

Q-34	**FinalMessTxt**<br>[QID 106344]<br>Text display [X]	1	This is the end of the questionnaire. By clicking the SUBMIT button you can send the data of the questionnaire. After this you can no longer make changes to the questionnaire.<br><br>**Question attribute** / **Value**<br>statistics_showgraph / 1

LimeSurvey
Version 2.6.7 Build SondagesPro 1.7.3
Like it? Donate to LimeSurvey
