# Supplementary material for: Challenges and opportunities for falls prevention: an online survey across European healthcare professionals
Source: Eur Geriatr Med. 2025 Jun 17;16(4):1269–82. doi: 10.1007/s41999-025-01237-5 (PMC12378296; doi:10.1007/s41999-025-01237-5)
Supplement: Supplementary file 2 — Supplementary file1 (DOCX 79 KB) [file 41999_2025_1237_MOESM2_ESM.docx]

Appendix

Supplementary Table 1. Baseline characteristics per region

|  | **Western (n=242)** | **Northern (n=487)** | **Eastern (n=563)** | **Southern (n=377)** |
| --- | --- | --- | --- | --- |
| **Age (median, IQR)** | **44 (35-54)** | **47 (39-57)** | **49 (40-58)** | **44 (34-53)** |
| **Gender (female)** | **68.2%** | **81.9%** | **74.1%** | **71.6%** |
| **Profession** |  |  |  |  |
| Physician | **45.0%** | **33.7%** | **37.7%** | **50.9%** |
| Physiotherapist | **22.3%** | **48.5%** | **44.5%** | **15.9%** |
| Nursing professionals across all levels | **2.9%** | **8.2%** | **10.0%** | **23.3%** |
| Occupational therapist | **17.8%** | **6.0%** | **5.2%** | **1.1%** |
| Other | **11.9%** | **3.7%** | **2.5%** | **8.8%** |
| **Type of physicians** |  |  |  |  |
| Practicing Geriatrician or specialist in care of older adults | **72.5%** | **75.6%** | **42.7%** | **57.8%** |
| Trainee Geriatrician or specialist in care of older adults | **2.8%** | **16.5%** | **12.6%** | **7.8%** |
| Non-practicing Geriatrician or specialist in care of older adults | **2.8%** | **3.0%** | **1.9%** | **3.6%** |
| GP | **8.3%** | **1.8%** | **34.0%** | **15.1%** |
| GP in training |  | **0.6%** | **2.4%** | **1.0%** |
| Other | **13.8%** | **2.4%** | **6.3%** | **14.6%** |
| **Health care working experience years (median, IQR)** | **17.5 (8-26)** | **20 (12-30)** | **21 (12-30)** | **18 (8-26.5)** |
| **Current work environment** |  |  |  |  |
| General practice or community setting | **9.1%** | **24.8%** | **44.3%** | **25.5%** |
| Hospital, mostly clinical ward | **21.1%** | **40.9%** | **16.5%** | **29.7%** |
| Long-term care facility or rehabilitation setting | **40.5%** | **11.1%** | **24.1%** | **20.4%** |
| Hospital, mostly outpatient clinic | **12.0%** | **11.5%** | **8.9%** | **12.7%** |
| Other | **17.4%** | **11.7%** | **6.1%** | **11.7%** |

Eastern Europe: Belarus, Bulgaria, Czech Republic, Hungary, Israel, Poland, Romania, Russia, Slovakia, Türkiye, Ukraine

Northern Europe: Denmark, Estonia, Finland, Iceland, Ireland, Latvia, Lithuania, Norway, Sweden, United Kingdom

Southern Europe: Albania, Andorra, Bosnia and Herzegovina, Croatia, Greece, Italy, Malta, Montenegro, North Macedonia, Portugal, San Marino, Serbia, Slovenia, Spain

Western Europe: Austria, Belgium, France, Germany, Luxembourg, Netherlands, Switzerland

Supplementary Table 2. Baseline characteristics per country with at least 50 participants

|  | **Israel (n=448)** | **UK (n=224)** | **Austria (n=120)** | **Slovenia (n=103)** | **Spain (n=76)** | **Malta (n=72)** | **Sweden (n=61)** |
| --- | --- | --- | --- | --- | --- | --- | --- |
| **Age (median, IQR)** | **50 (41-58) (n=424)** | **49 (42.5-57)** | **41 (32-50)** | **40 (32-49.5)** | **46 (34.25-55)** | **38 (30-52)** | **51 (39.5-58.5)** |
| **Gender (female)** | **74.8% (n=441)** | **80.8%** | **66.7%** | **91.3%** | **68.4%** | **72.2%** | **83.6%** |
| **Profession** |  |  |  |  |  |  |  |
| Physician | **23.6%** | **30.4%** | **25.0%** | **8.7%** | **90.8%** | **29.2%** | **19.7%** |
| Physiotherapist | **55.4%** | **53.6%** | **31.7%** | **26.2%** | **2.6%** | **37.5%** | **78.7%** |
| Nursing professionals across all levels | **11.7%** | **5.8%** | **0.8%** | **54.4%** | **3.9%** | **19.4%** |  |
| Occupational therapist | **6.5%** | **5.8%** | **35.8%** |  |  | **4.2%** | **1.6%** |
| Other | **2.7%** | **4.4%** | **5.8%** | **10.7%** | **2.6%** | **9.7%** |  |
| **Type of physicians** |  |  |  |  |  |  |  |
| Practicing Geriatrician or specialist in care of older adults | **29.0%** | **85.3%** | **26.7%** | **33.3%** | **81.2%** | **52.4%** | **66.7%** |
| Trainee Geriatrician or specialist in care of older adults | **3.0%** | **5.9%** | **3.3%** |  | **5.8%** | **42.9%** |  |
| Non-practicing Geriatrician or specialist in care of older adults | **1.0%** | **1.5%** |  |  |  |  | **16.7%** |
| GP | **58.0%** | **4.4%** | **23.3%** | **66.7%** |  |  |  |
| GP in training | **2.0%** | **1.5%** |  |  | **1.4%** |  |  |
| Other | **7.0%** | **1.5%** | **46.7%** |  | **11.6%** | **4.8%** | **16.7%** |
| **Health care working experience years (median, IQR)** | **22 (12-32) (n=403)** | **24 (16.25-31)** | **16 (6-25)** | **17 (6.5-27)** | **18 (7-24.5)** | **11 (8-25)** | **23 (13.5-29.5)** |
| **Current work environment** |  |  |  |  |  |  |  |
| General practice or community setting | **52.4%** | **34.4%** | **5.0%** | **49.5%** | **6.6%** | **12.5%** | **27.9%** |
| Hospital, mostly clinical ward | **13.2%** | **41.5%** | **5.8%** | **6.8%** | **52.6%** | **27.8%** | **14.8%** |
| Long-term care facility or rehabilitation setting | **25.5%** | **3.1%** | **67.5%** | **22.3%** | **11.8%** | **31.9%** | **37.7%** |
| Hospital, mostly outpatient clinic | **3.1%** | **11.6%** | **1.7%** | **1.0%** | **22.4%** | **19.4%** | **6.6%** |
| Other | **5.9%** | **9.4%** | **20.0%** | **20.4%** | **6.6%** | **8.3%** | **13.1%** |

Supplementary Table 3. Education and knowledge per region

|  | **Western (n=242*)** | **Northern (n=487*)** | **Eastern (n=563*)** | **Southern (n=377*)** |
| --- | --- | --- | --- | --- |
| **Knowledge on falls prevention** |  |  |  |  |
| Very knowledgeable | 48.8% | 65.9% | 59.4% | 38.2% |
| Some knowledge | 45.0% | 32% | 37.9% | 52.3% |
| Little knowledge | 6.2% | 1.8% | 2.7% | 8.5% |
| No knowledge |  | 0.2% |  | 1.1% |
| **Undergraduate education adequately prepared for clinical practice (agree/strongly agree)** | 29.9% | 17.5% | 34.2% | 26.6% |
| **Received education (yes)** | 68.5% | 89.3% | 83.1% | 79.3% |
| **Hours of education or training in last 5 years** |  |  |  |  |
| **Less than 5 hours** | 41.9% | 26.1% | 42.7% | 39.3% |
| **5 to 15 hours** | 27.4% | 34.2% | 35.1% | 33.2% |
| **More than 15 hours** | 30.7% | 39.7% | 22.2% | 27.6% |

Eastern Europe: Belarus, Bulgaria, Czech Republic, Hungary, Israel, Poland, Romania, Russia, Slovakia, Türkiye, Ukraine

Northern Europe: Denmark, Estonia, Finland, Iceland, Ireland, Latvia, Lithuania, Norway, Sweden, United Kingdom

Southern Europe: Albania, Andorra, Bosnia and Herzegovina, Croatia, Greece, Italy, Malta, Montenegro, North Macedonia, Portugal, San Marino, Serbia, Slovenia, Spain

Western Europe: Austria, Belgium, France, Germany, Luxembourg, Netherlands, Switzerland

***N** represents the first item in the table and may vary slightly between different entries

Supplementary Table 4. Education and knowledge per country with at least 50 participants

|  | **Israel (n=442*)** | **UK (n=224*)** | **Austria (n=120*)** | **Slovenia (n=103*)** | **Spain (n=76*)** | **Malta (n=72*)** | **Sweden (n=61*)** |
| --- | --- | --- | --- | --- | --- | --- | --- |
| **Knowledge on falls prevention** |  |  |  |  |  |  |  |
| Very knowledgeable | 61.8% | 78.1% | 35% | 24.3% | 57.9% | 47.2% | 55.7% |
| Some knowledge | 35.7% | 21.9% | 53.3% | 62.1% | 38.2% | 51.4% | 42.6% |
| Little knowledge | 2.5% |  | 11.7% | 12.6% | 3.9% | 1.4% | 1.6% |
| No knowledge |  |  |  | 1.0% |  |  |  |
| **Undergraduate education adequately prepared for clinical practice (agree/strongly agree)** | 35.9% | 11.2% | 31.6% | 32.1% | 14.4% | 29.2% | 11.5% |
| **Received education (yes)** | 85.3% | 96% | 47.5% | 74.8% | 93.4% | 93.1% | 86.7% |
| **Hours of education or training in last 5 years** |  |  |  |  |  |  |  |
| **Less than 5 hours** | 41.1% | 15.2% | 61.7% | 46.6% | 18.4% | 36.1% | 43.3% |
| **5 to 15 hours** | 36.0% | 33.9% | 23.3% | 42.7% | 26.3% | 36.1% | 40.0% |
| **More than 15 hours** | 22.9% | 50.9% | 15.0% | 10.7% | 55.3% | 27.8% | 16.7% |

***N** represents the first item in the table and may vary slightly between different entries

Supplementary Table 5. Education and knowledge per healthcare professional

|  | **Physicians (n=675*)** | **Physiotherapists (n=598*)** | **Others**  **(n=390*)** |
| --- | --- | --- | --- |
| **Knowledge on falls prevention** |  |  |  |
| Very knowledgeable | **53.8%** | **62.2%** | **45.9%** |
| Some knowledge | 40.6% | 35.6% | 47.7% |
| Little knowledge | 5.3% | 2.0% | 5.9% |
| No knowledge | 0.3% | 0.2% | 0.5% |
| **Undergraduate education adequately prepared for clinical practice (agree/strongly agree)** | 17.2% | 35.4% | 30.8% |
| **Received education (yes)** | 81.5% | 87.4% | 74.4% |
| **Hours of education or training in last 5 years** |  |  |  |
| **Less than 5 hours** | 35.2% | 33.1% | 44.6% |
| **5 to 15 hours** | 31.0% | 36.7% | 31.6% |
| **More than 15 hours** | 33.8% | 30.2% | 23.8% |

***N** represents the first item in the table and may vary slightly between different entries

Supplementary Table 6. Current falls prevention approaches and practice per region

|  | **Western (n=183*)** | **Northern (n=376*)** | **Eastern (n=430*)** | **Southern (n=262*)** |
| --- | --- | --- | --- | --- |
| **Opportunistic screening during consultations (often/always)** | 76.5% | 88.3% | 70.0% | 66.8% |
| 1. History of falls | 89.0% | 92.0% | 92.6% | 74.0% |
| 1. Felt unsteady when walking or standing | 92.2% | 92.0% | 88.1% | 78.3% |
| 1. Patient expresses worries about walling | 85.6% | 84.4% | 78.7% | 78% |
| **When patient reported falling, the following components were considered often/ always** | | | | |
| 1. Whether injured | 91.7% | 90.7% | 93.7% | 82.6% |
| 1. Multiple falls | 93.3% | 92.9% | 95.3% | 85.9% |
| 1. Unable to get up after falling | 82.3% | 86.2% | 82% | 75.7% |
| 1. Whether fall was accompanied by suspected loss of consciousness | 80.1% | 87.3% | 83.2% | 68.3% |
| 1. Frailty | 84.5% | 82.9% | 72.4% | 75.3% |
| **Shared decision making often/always)** | | | | |
| Inquiring about older adults' thoughts on falls as a part of comprehensive fall risk assessment | 74.6% | 76.6% | 74.4% | 67.8% |
| Helping the patient to explore and compare treatment options | 69.5% | 66.1% | 66.6% | 66.3% |
| Considering patient’s or caregiver’s preferences and goals when developing care plans | 87% | 84.9% | 86.4% | 70.1% |
| Reaching collective decisions with patients | 85.9% | 84.6% | 81.1% | 68.2% |
| **Strategies to increase to adherence (often/always)** | | | | |
| Motivational interview techniques | 73.0% | 45.4% | 69.1% | 52.4% |
| Sharing patient materials | 40.4% | 41% | 47.2% | 27.7% |
| Organizing follow-up visits | 45.5% | 45.4% | 38.8% | 54.0% |

Eastern Europe: Belarus, Bulgaria, Czech Republic, Hungary, Israel, Poland, Romania, Russia, Slovakia, Türkiye, Ukraine

Northern Europe: Denmark, Estonia, Finland, Iceland, Ireland, Latvia, Lithuania, Norway, Sweden, United Kingdom

Southern Europe: Albania, Andorra, Bosnia and Herzegovina, Croatia, Greece, Italy, Malta, Montenegro, North Macedonia, Portugal, San Marino, Serbia, Slovenia, Spain

Western Europe: Austria, Belgium, France, Germany, Luxembourg, Netherlands, Switzerland

**N** represents the first item in the table and may vary slightly between different entries

Supplementary Table 7. Current falls prevention approaches and practice per country with at least 50 participants

|  | **Israel (n=333*)** | **UK (n=194*)** | **Austria (n=89*)** | **Slovenia (n=61*)** | **Spain (n=57*)** | **Malta (n=47*)** | **Sweden (n=38*)** |
| --- | --- | --- | --- | --- | --- | --- | --- |
| **Opportunistic screening during consultations (often/always)** | 67.8% | 90.2% | 67.4% | 70.5% | 75.4% | 68.1% | 81.5% |
| 1. History of falls | 94.0% | 94.9% | 85% | 67.2% | 82.4% | 85.1% | 79.5% |
| 1. Felt unsteady when walking or standing | 91.0% | 94.4% | 95.4% | 76.5% | 84.3% | 91.3% | 92.1% |
| 1. Patient expresses worries about walling | 78.0% | 91.7% | 89.7% | 81.3% | 86% | 80.5% | 81.6% |
| **When patient reported falling, the following components were considered often/always** | | | | | | | |
| 1. Whether injured | 94.6% | 92.2% | 89.4% | 81.0% | 93.0% | 91.5% | 79% |
| 1. Multiple falls | 95.8% | 94.8% | 92.0% | 78.1% | 94.8% | 97.9% | 89.5% |
| 1. Unable to get up after falling | 82.1% | 91.3% | 80.5% | 65.6% | 87.7% | 95.7% | 76.3% |
| 1. Whether fall was accompanied by suspected loss of consciousness | 80.6% | 90.7% | 70.1% | 64.0% | 93.0% | 84.7% | 71.1% |
| 1. Frailty | 69.5% | 89.7% | 80.4% | 68.7% | 86.0% | 86.9% | 76.3% |
| **Shared decision making (often/always)** | | | | | | | |
| Inquiring about older adults' thoughts on falls as a part of comprehensive fall risk assessment | 72.2% | 77.6% | 70.3% | 71.0% | 79.0% | 73.9% | 72.9% |
| Helping the patient to explore and compare treatment options | 62.7% | 73.4% | 66.7% | 71.0% | 79.0% | 62.8% | 35.1% |
| Considering patient’s or caregiver’s preferences and goals when developing care plans | 86.7% | 88.6% | 85.8% | 62.9% | 80.7% | 84.8% | 81% |
| Reaching collective decisions with patients | 80.3% | 88.0% | 86.9% | 59.6% | 82.4% | 80.5% | 78.4% |
| **Strategies to increase to adherence (often/always)** | | | | | | | |
| Motivational interview techniques | 70.7% | 43.5% | 88.2% | 64.5% | 61.4% | 46.7% | 32.4% |
| Sharing patient materials | 48.5% | 46.1% | 31.8% | 32.3% | 26.3% | 22.2% | 24.3% |
| Organizing follow-up visits | 37.5% | 64.6% | 38.8% | 45.1% | 56.2% | 84.8% | 62.1% |

***N** represents the first item in the table and may vary slightly between different entries

Supplementary Table 8. Current falls prevention approaches and practice per health care professional

|  | **Physicians (n=542*)** | **Physiotherapist (n=462*)** | **Others (n=247*)** |
| --- | --- | --- | --- |
| **Opportunistic screening during consultations (often/always)** | 75.3% | 80.5% | 68.0% |
| 1. History of falls | 87.5% | 82.6% | 79.5% |
| 1. Felt unsteady when walking or standing | 85.3% | 93.9% | 82.2% |
| 1. Patient expresses worries about walling | 79.0% | 88.7% | 75.8% |
| **When patient reported falling, the following components were considered often/always** | | | |
| 1. Whether injured | 90.8% | 92.4% | 84.5% |
| 1. Multiple falls | 93.8% | 94.3% | 75.5% |
| 1. Unable to get up after falling | 84.0% | 83.4% | 65.2% |
| 1. Whether fall was accompanied by suspected loss of consciousness | 92.1% | 77.3% | 73.1% |
| 1. Frailty | 83.3% | 72.8% | 75.9% |
| **Shared decision making (often/always)** | | | |
| Inquiring about older adults' thoughts on falls as a part of comprehensive fall risk assessment | 72.7% | 74.2% | 74.7% |
| Helping the patient to explore and compare treatment options | 64.8% | 70.1% | 64.5% |
| Considering patient’s or caregiver’s preferences and goals when developing care plans | 80.9% | 89.1% | 73.9% |
| Reaching collective decisions with patients | 79.5% | 85.1% | 72.7% |
| **Strategies to increase to adherence (often/always)** | | | |
| Motivational interview techniques | 51.6% | 65.2% | 62.3% |
| Sharing patient materials | 46.4% | 34.5% | 40.1% |
| Organizing follow-up visits | 48.4% | 51.7% | 45.9% |

***N** represents the first item in the table and may vary slightly between different entries

Supplementary Table 9. Current falls prevention approaches and practice per working environment

|  | **General practice or community setting (n=365*)** | **Hospital, mostly clinical ward (n=346*)** | **Long-term care facility or rehabilitation setting (n=250*)** | **Hospital, mostly outpatient clinic (n=153*)** | **Other (n=127*)** |
| --- | --- | --- | --- | --- | --- |
| **Opportunistic screening during consultations (often/always)** | 71.0% | 81.3% | 73.6% | 82.3% | 70.1% |
| 1. History of falls | 87.0% | 91.4% | 88.8% | 90.2% | 76.5% |
| 1. Felt unsteady when walking or standing | 86.4% | 90.5% | 92.2% | 84.2% | 79.7% |
| 1. Patient expresses worries about walling | 79.8% | 85.3% | 85.4% | 81.0% | 74.2% |
| **When patient reported falling, the following components were considered often/always** | | | | | |
| 1. Whether injured | 90.2% | 91.4% | 91.7% | 90.2% | 82.8% |
| 1. Multiple falls | 91.3% | 94.9% | 93.9% | 94.1% | 76.6% |
| 1. Unable to get up after falling | 85.0% | 85.9% | 80.5% | 82.2% | 65.3% |
| 1. Whether fall was accompanied by suspected loss of consciousness | 83.1% | 89.0% | 79.2% | 88.8% | 66.1% |
| 1. Frailty | 73.9% | 82.4% | 79.8% | 84.2% | 66.6% |
| **Shared decision making (often/always)** | | | | | |
| Inquiring about older adults' thoughts on falls as a part of comprehensive fall risk assessment | 74.0% | 75.3% | 74.4% | 76.0% | 62.8% |
| Helping the patient to explore and compare treatment options | 68.8% | 63.7% | 64.5% | 76.7% | 60.3% |
| Considering patient’s or caregiver’s preferences and goals when developing care plans | 81.0% | 84.5% | 84.6% | 86.0% | 70.8% |
| Reaching collective decisions with patients | 79.7% | 82.2% | 77.9% | 86.0% | 72.5% |
| **Strategies to increase to adherence (often/always)** | | | | | |
| Motivational interview techniques | 62.1% | 50.0% | 66.3% | 60.0% | 53.4% |
| Sharing patient materials | 44.1% | 37.5% | 19.3% | 54.7% | 37.8% |
| Organizing follow-up visits | 53.0% | 41.1% | 40.7% | 58.7% | 48.4% |

***N** represents the first item in the table and may vary slightly between different entries

Supplementary Table 10. Top-5 Challenges experienced in implementing fall prevention per region: Based on Combined "Extremely" or "Very Challenging" Ratings

|  | **Western (n=141*)** | **Northern (n=269*)** | **Eastern (n=304*)** | **Southern (n=179*)** |
| --- | --- | --- | --- | --- |
| Staffing issues | **2*** | **1** | **3** | **2** |
| A lack of time | **1** | **2** | **2** | **1** |
| Older adults’ noncompliance | **2*** | **5** | **1** | **5** |
| Workload related to fall prevention | **5** | **3** |  | **3** |
| Prioritizing other tasks | **4** | **4** | **4** |  |
| A lack of personal involvement in the development process of local falls prevention program |  |  |  | **4** |
| Conflict with older adult/family or with their expectations |  |  | **5** |  |

Eastern Europe: Belarus, Bulgaria, Czech Republic, Hungary, Israel, Poland, Romania, Russia, Slovakia, Türkiye, Ukraine

Northern Europe: Denmark, Estonia, Finland, Iceland, Ireland, Latvia, Lithuania, Norway, Sweden, United Kingdom

Southern Europe: Albania, Andorra, Bosnia and Herzegovina, Croatia, Greece, Italy, Malta, Montenegro, North Macedonia, Portugal, San Marino, Serbia, Slovenia, Spain

Western Europe: Austria, Belgium, France, Germany, Luxembourg, Netherlands, Switzerland

***N** represents the first item in the table and may vary slightly between different entries

Supplementary Table 11. Top-5 Challenges experienced in implementing fall prevention per country with at least 50 participants: Based on Combined "Extremely" or "Very Challenging" Ratings

|  | **Israel (n=237*)** | **UK (n=148*)** | **Austria (n=63*)** | **Slovenia (n=35*)** | **Spain (n=41*)** | **Malta (n=29*)** | **Sweden (n=27*)** |
| --- | --- | --- | --- | --- | --- | --- | --- |
| Staffing issues | **3** | **1** | **2** | **3** | **1*** | **1*** | **4** |
| A lack of time | **2** | **2** | **1** | **1** | **1*** | **1*** | **1** |
| Older adults’ noncompliance | **1** | **5** | **3** | **5*** |  | **3*** | **5*** |
| Workload related to fall prevention |  | **3** | **5** |  | **1*** |  | **2*** |
| Prioritizing other tasks | **4** | **4** | **4** |  |  | **5** | **2*** |
| A lack of educational structures |  |  |  |  | **5*** |  | **5*** |
| Poor communication and information sharing |  |  |  |  |  |  | **5*** |
| Conflict with older adult/family or with their expectations | **5** |  |  |  |  | **3*** |  |
| Feeling helpless, frustrated, or concerned about ability to control falls management |  |  |  | **4** |  |  |  |
| Limited knowledge and skills about falls prevention |  |  |  | **5*** |  |  |  |
| A lack of personal involvement in the development process of local falls prevention program |  |  |  | **2** |  |  |  |
| A lack of quality improvement structures |  |  |  |  | **5*** |  |  |

***N** represents the first item in the table and may vary slightly between different entries

Supplementary Table 12. Top-5 Challenges experienced in implementing fall prevention per health care professional: Based on Combined "Extremely" or "Very Challenging" Ratings

|  | **Physicians (n=414*)** | **Physiotherapists (n=324*)** | **Others (n=154*)** |
| --- | --- | --- | --- |
| Staffing issues | **2** | **3** | **2** |
| A lack of time | **1** | **2** | **1** |
| Older adults’ noncompliance | **3** | **1** | **3** |
| Workload related to fall prevention | **5** | **5** | **5** |
| Prioritizing other tasks | **4** | **4** | **4** |

***N** represents the first item in the table and may vary slightly between different entries

Supplementary Table 13. Top-5 facilitators for further falls prevention activities per region

|  | **Western** | **Northern** | **Eastern** | **Southern** |
| --- | --- | --- | --- | --- |
| More time | **4** | **2** | **1** | **3** |
| Easy to use guideline | **1** |  | **2** | **2** |
| Sufficient resources | **2** | **1** |  |  |
| Increased education and training | **5** | **4** |  | **1** |
| Increased collaboration | **3** | **3** | **4** | **4** |
| Programs raising awareness and interest |  |  | **3** |  |
| Implementation guides of falls prevention |  |  | **5** | **5** |
| Improved information sharing between professionals |  | **5** |  |  |

Eastern Europe: Belarus, Bulgaria, Czech Republic, Hungary, Israel, Poland, Romania, Russia, Slovakia, Türkiye, Ukraine

Northern Europe: Denmark, Estonia, Finland, Iceland, Ireland, Latvia, Lithuania, Norway, Sweden, United Kingdom

Southern Europe: Albania, Andorra, Bosnia and Herzegovina, Croatia, Greece, Italy, Malta, Montenegro, North Macedonia, Portugal, San Marino, Serbia, Slovenia, Spain

Western Europe: Austria, Belgium, France, Germany, Luxembourg, Netherlands, Switzerland

Supplementary Table 14. Top-5 facilitators for further falls prevention activities per country with at least 50 participants

|  | **Israel** | **UK** | **Austria** | **Slovenia** | **Spain** | **Malta** | **Sweden** |
| --- | --- | --- | --- | --- | --- | --- | --- |
| More time | **1** | **2** | **4*** |  | **1** | **4*** |  |
| Easy to use guideline | **2** |  | **1** | **2** |  | **1*** |  |
| Sufficient resources |  | **1** | **3** |  | **2** |  | **2*** |
| Increased education and training |  |  | **2** | **1** |  | **1*** | **1** |
| Increased collaboration |  | **3** |  |  | **3*** | **4*** |  |
| Patients empowerment to falls prevention |  | **5** |  |  |  | **3** |  |
| Implementation guides of falls prevention | **5** |  |  |  |  | **4*** | **4*** |
| Increased collaboration |  |  | **4*** | **3** |  |  |  |
| Improved information sharing between professionals | **4** | **4** |  | **4** |  |  |  |
| A staff member focusing on implementation of falls prevention |  |  |  |  | **5** |  |  |
| Programs raising awareness and interest | **3** |  |  | **5** | **3*** |  |  |
| Motivated collegues |  |  |  |  |  |  | **4*** |
| Programs involving and empowering staff |  |  |  |  |  |  | **2*** |

Supplementary Table 15. Top-5 facilitators for further falls prevention activities per health care professional

|  | **Physicians** | **Physiotherapists** | **Others** |
| --- | --- | --- | --- |
| More time | **1** | **1** | **4** |
| Easy to use guideline | **4** | **2** | **2** |
| Sufficient resources | **2** |  | **5** |
| Increased education and training | **5** | **5** | **1** |
| Increased collaboration | **3** | **4** |  |
| Improved information sharing between professionals |  | **3** | **3** |
